# Effects of weight loss and weight gain on HbA1c, systolic blood pressure and total cholesterol in three subgroups defined by blood glucose: a pooled analysis of two behavioural weight management trials in England

**DOI:** 10.1136/bmjopen-2024-095046

**Published:** 2025-04-15

**Authors:** Katharine Pidd, Penny Breeze, Amy Ahern, Simon J Griffin, Alan Brennan

**Affiliations:** 1 School of Medicine and Population Health, The University of Sheffield, Sheffield, UK; 2 MRC Epidemiology Unit, University of Cambridge, Cambridge, UK

**Keywords:** Diabetes Mellitus, Type 2, Body Mass Index, Obesity

## Abstract

**Abstract:**

**Objectives:**

To estimate the association between weight and cardiometabolic risk factors across subgroups of individuals with normoglycaemia, non-diabetic hyperglycaemia and type 2 diabetes (T2D) and to explore whether the association differs between weight loss and weight gain.

**Design:**

Observational analysis using mixed-effects regression models of pooled trial data.

**Participants:**

The Weight loss Referral for Adults in Primary care (n=1267) and Glucose Lowering through Weight management (n=577) trials recruited individuals with overweight or obesity (body mass index, BMI >25 kg/m^2^) from primary care practices across England.

**Primary and secondary outcome measures:**

The primary outcome measures were the relationships between a change in (BMI; kg/m^2^) and a change in glycated haemoglobin (HbA_1c_; mmol/mol), total cholesterol (mmol/L) or systolic blood pressure (SBP; mm Hg) across three subgroups of individuals with: normoglycaemia, non-diabetic hyperglycaemia and T2D. Secondary outcomes included the influence of weight loss versus weight gain on these relationships.

**Results:**

HbA_1c_ is positively related to a change in BMI, and a 1 kg/m^2^ change was related to a 1.5 mmol/mol (95% CI: 1.1 to 1.9) change in HbA_1c_ in individuals with T2D, 0.6 mmol/mol (95% CI: 0.4 to 0.8) change in those with non-diabetic hyperglycaemia and 0.3 mmol/mol (95% CI: 0.2 to 0.4) change in those with normoglycaemia. In individuals with normoglycaemia, weight gain has a larger impact on HbA_1c_ than weight loss, with a 0.5 mmol/mol (95% CI: 0.3 to 0.7) increase per 1 kg/m2 gained, compared with a relationship that is 0.3 mmol/mol smaller (95% CI: −0.6 to −0.1) per 1 kg/m^2^ of weight loss. BMI reduction improved SBP and total cholesterol significantly; however, effects did not differ between the three subgroups.

**Conclusions:**

Cardiometabolic risk factors are associated with changes in weight. The association with HbA_1c_ varies by diabetes status, with increasing magnitude in those with non-diabetic hyperglycaemia and T2D. Weight gain has a larger impact on HbA_1c_ than weight loss in individuals with normoglycaemia, implying an asymmetric relationship.

**Strengths and limitations of this study**

- This analysis applied a linear mixed-effects regression approach allowing for individual variability.
- The study sample was large, pooled from two trials with a similar setup and interventions, allowing comparison of the relationships by type 2 diabetes status.
- The results were robust to missing data, trial differences and medication use.
- The relationships between weight and cardiometabolic risk presented here may not generalise to alternative weight loss approaches.
- Data availability prevented analysis of the relationship with other metabolic risk factors that may be of importance.

## Introduction

 Obesity is a significant risk factor for many comorbidities, including cardiovascular disease (CVD) and type 2 diabetes (T2D). Weight loss has been linked to reduced risk of CVD and T2D through improvements in blood glucose, blood pressure and cholesterol levels.^1^ T2D is a metabolic condition characterised by insulin resistance, whereby the body cannot effectively produce or use insulin to manage blood glucose levels, resulting in hyperglycaemia.^2^ Hyperglycaemia causes inflammation and cell damage, leading to macro and microvascular complications.^3^ Research suggests that glycaemic control improves with weight loss.^14^,^10^ However, the magnitude of this relationship appears less consistent. An analysis found it to only exist in cases of larger weight loss,^6^ while others found it significant across all magnitudes of weight loss.^5 7^ Additionally, some found the relationship to diminish over time despite the weight loss remaining.^5 8^ Much of this research was completed on individuals with T2D, but the relationship between weight loss and glycated haemoglobin (HbA_1c_), varies depending on T2D status.^1^

Previous studies have not differentiated between risk levels for T2D when analysing individuals without T2D. In the UK, those at high risk of developing T2D are defined by having HbA_1c_ between 42 mmol/mol and 48 mmol/mol and are reported to have non-diabetic hyperglycaemia (NDH).^11^ While individuals within this range do not meet the diagnosis criteria of T2D, they may have an altered metabolic response to weight loss than individuals without hyperglycaemia. Weight loss can improve glucose control and diabetes risk within individuals with NDH,^12^ with some suggestion that their starting weight may relate to the improvements seen.^13^ However, the magnitude of the relationship with HbA_1c_ has not been ascertained.

High blood pressure and cholesterol have been related to T2D through complex multidirectional pathophysiological mechanisms. Individuals with hypertension can exhibit insulin resistance^3^ and high cholesterol levels inhibit insulin secretion through beta-cell dysfunction.^14^ Diabetes can trigger hypertension by promoting sodium retention^15^ and decreasing the efficiency of cholesterol absorption.^16^ Furthermore, certain pharmacological treatments for hypertension and hypercholesterolaemia have been linked to reduced glycaemic control, and in some cases, new onset of T2D.^17 18^

Weight loss was found to reduce cholesterol levels and systolic blood pressure (SBP) for individuals with insulin resistance or T2D;^9 19^ however, this can vary with the type of weight loss intervention.^20^ A trial analysis found that a 1 kg weight change related to a 0.4 mm Hg change in SBP and a 0.02 mmol/L change in total cholesterol levels when considering behavioural weight management programmes for individuals with overweight/obesity.^1^ However, these relationships were not investigated by T2D status, despite the interrelated metabolic pathways.

Few analyses have compared these relationships between individuals without T2D and with T2D, and fewer have considered individuals with NDH. The aims of this study were to estimate the relationship between weight loss and metabolic risk factors including HbA_1c_, blood pressure and cholesterol levels; to detect differences in these associations by glycaemia status and investigate how the magnitude and direction of weight change, medication use and baseline body mass index (BMI) affect these relationships. To investigate these aims, regression techniques were performed on data pooled from two trials.

## Methods

### The data

Data from two behavioural weight management randomised controlled trials (RCTs) were pooled to form the sample. The Weight loss Referral for Adults in Primary care (WRAP) trial recruited adults with a BMI of 28 kg/m^2^ or higher from primary care practices and randomly assigned them to receive brief advice and self-help materials, or a group-based behavioural weight-management programme delivered by WW (formerly Weight Watchers), for either 12 or 52 weeks.^21 22^ For this analysis, we used follow-up data collected at 12 and 60 months.

The Glucose Lowering through Weight management (GLoW) trial recruited adults with a BMI of 25 kg/m^2^ or more, with a diagnosis of T2D within the last 3 years from primary care practices.^23 24^ Participants were randomly assigned to either an NHS structured diabetes education programme or a minimally tailored diabetes education delivered by telephone and a 6-month behavioural weight management programme delivered by WW. Follow-up data at 6 and 12 months were used. The trials collected demographic information, BMI (kg/m^2^), HbA_1c_ (mmol/mol), SBP (mm Hg) and total cholesterol (mmol/l) levels. Participants were asked to complete self-reported questionnaires, and anthropometric measurements and blood samples were taken by trained staff at measurement appointments. Individuals not able to attend a measurement appointment were requested to provided self-reported weight. Participants’ informed consent was obtained in both trials. More information on these trials is reported elsewhere.^21^,^23^

The sample population was divided into three subgroups: those with normoglycaemia, those with NDH and those with T2D. Participants with HbA_1c_ below 42 mmol/mol at baseline, without a recorded diagnosis of T2D were defined as not having T2D and having normoglycaemia (non-diabetic normoglycaemia, NDN). Participants with a recorded diagnosis of T2D or HbA_1c_ at 48 mmol/mol or higher at baseline were defined as having T2D. Any individual without a recorded diagnosis of T2D, with an HbA_1c_ of at least 42 mmol/mol and less than 48 mmol/mol were assigned to the NDH group.

### Statistical approach

The mean changes in risk factors at each time point are reported in online supplemental table S2. The difference in the mean changes between subpopulations was evaluated using a Welch t-test. The primary analysis investigated how a change in BMI related to a change in HbA_1c_ (mmol/mol), total cholesterol (mmol/L) or SBP (mm Hg) using a linear mixed-effects regression approach for each subgroup. The dependent variable described change in BMI from baseline to each follow-up point at 6, 12 and 60 months and the independent variables described change in the cardiometabolic risk over the corresponding time point, from baseline to 6, 12 and 60 months. Control variables included within the regression as fixed effects were age at baseline, sex, ethnicity, BMI at baseline, baseline observation for the independent variable, time point and trial sample for all regressions. The regressions allow variation in the intercept by participant. The regression specification is:


$$\begin{array}{l} {\left(Change\;in\;risk\;factor\;from\;baseline\;to\;timepoint\;t\right)}_{1}=\\ \beta_{0}+u_{i}+\beta_{1}\times {\left(Change\;in\;BMI\;from\;baseline\;to\;time\;pointt\right)}_{i}+\\ \beta_{2}\times \left(t=60\;months\;or\;t=6\;months\right)+Control\;Variables+\epsilon_{it} \end{array}$$

where *i* represents the individual, *t* represents the time points at 6, 12 and 60 months. Between-individual error is represented by *u_i_*, while *ε_it_* represents overall residual error, both assumed to be normally distributed with a mean of zero. The coefficient of interest, *β_1_*, represents the change in the risk factor level associated with a 1 kg/m^2^ change in BMI. A separate regression model was performed for each subgroup, and a Welch t-test compared coefficients between the subgroups to accommodate different variances. Statistical significance was assumed at the 95% CI. A complete case analysis was used, assuming that data were missing completely at random. Analyses were conducted using computer package R V.4.2.1.

Additional analyses considered the difference between the relationship between BMI and HbA_1c_, cholesterol and SBP depending on the direction or magnitude of weight change. To investigate how the relationships change when considering weight loss vs weight gain, an interaction variable between BMI change and an indicator of the direction of BMI change was included within the regressions. To investigate the relationship with HbA_1c_ between individuals who have small weight loss (>0% and <5%) and individuals with moderate weight loss (≥5%), the base regression model was performed separately for these two subpopulations. In addition, the regressions were completed with HbA_1c_ converted from mmol/mol to per cent (DCCT). The equation used to convert HbA_1c_ units, and the results are in online supplemental tables S7 and S11.

To evaluate the impact of medication use, additional medication indicators were added to the model. This analysis, performed on the GLoW trial population of individuals with T2D, expanded on the base regression model for each metabolic measure, HbA_1c_, SBP or cholesterol by adding medication use indicators for glucose-lowering, anti-hypertensive or cholesterol-lowering medications.

Finally, an exploratory analysis considered the impact of baseline BMI on the relationship between weight change and HbA_1c_ by dividing the pooled population by BMI classifications overweight (≥25 kg/m^2^ and <30 kg/m^2^), obese (≥30 kg/m^2^ and <40 kg/m^2^) and severe obesity (≥40 kg/m2). The base regression model relating BMI change to HbA_1c_ change is applied for each subgroup.

### Sensitivity analyses

To assess the impact of missing data on regression outputs, incomplete variables were imputed for individuals with recorded baseline HbA_1c_ using multiple imputation with chained equations for the primary analysis. All variables in the analyses were included in the imputation. Further description of this process is in online supplemental figure S1.

An additional regression with a diabetes status indicator and interaction term with BMI change was performed on the pooled population to see how the relationship changes between subgroups.

To investigate trial differences in the relationship across the T2D population, the base regression analysis between BMI and HbA_1c_ was performed on the WRAP and GLoW populations separately, and the regression outputs compared.

Unadjusted regression models were performed for the primary analysis between BMI change and HbA_1c_, cholesterol and SBP.

### Patient and public involvement

Patients or the public were not directly involved in the analysis presented in this paper. Patient and public engagement panels were involved in both the GLoW and WRAP trials that collected the data used within this analysis.

## Results

### Study sample

Baseline characteristics for the trials are reported in table 1. In both trials, over 80% of participants were white, and the majority were women. The proportion of individuals by Index of Multiple Deprivation was unbalanced between the trials; however, it was balanced in the combined sample. The average baseline BMI and SBP did not differ between the trials; however, average HbA_1c_ was higher in the GLoW trial, due to differences in inclusion criteria, and average cholesterol was higher in the WRAP trial. Baseline characteristics presented by T2D status subgroups are presented in online supplemental table S1.

**Table 1:** Baseline statistics by trial population and subgroups: normoglycaemia, non-diabetic hyperglycaemia and type 2 diabetes

			AllN=1844	WRAPN=1267	GLoWN=577	P value of trial difference
Age	Baseline age (years)	Mean (SD)	55 (14)	53 (14)	60 (13)	**<0.01**
	Missing	N (%)	0 (0)	0 (0)	0 (0)	
Sex	Women	N (%)	1137 (62)	859 (68)	301 (52)	**<0.01**
	Men	N (%)	655 (36)	408 (32)	224 (39)	
	Missing	N (%)	52 (3)	0 (0)	52 (9)	
Ethnicity	White	N (%)	1610 (87)	1136 (90)	474 (82)	**<0.01**
	Missing	N (%)	44 (2)	44 (4)	0 (0)	
Diabetes status	No type 2 diabetes	N (%)	564 (31)	564 (45)	0 (0)	
	Type 2 diabetes	N (%)	767 (42)	190 (15)	577 (100)	
	High risk of type 2 diabetes	N (%)	136 (7)	136 (11)	0 (0)	
	Missing	N (%)	377 (20)	377 (30)	0 (0)	
IMD Score	1—Most deprived	N (%)	423 (23)	344 (27)	79 (14)	**<0.01**
	2	N (%)	406 (22)	325 (26)	81 (14)	**<0.01**
	3	N (%)	391 (21)	267 (21)	124 (22)	0.16
	4	N (%)	287 (24)	174 (14)	113 (20)	**<0.01**
	5—Least deprived	N (%)	268 (25)	155 (12)	113 (20)	**<0.01**
	Missing	N (%)	69 (4)	2 (0)	67 (12)	
Baseline BMI	BMI (kg/m^2^)	Mean (SD)	34.6 (5.7)	34.5 (5.1)	34.6 (6.8)	0.81
	Missing	N (%)	2 (0)	0 (0)	2 (0)	
Baseline HbA_1c_	HbA_1c_ (mmol/mol)	Mean (SD)	46 (13)	41 (10)	54 (14)	**<0.01**
	HbA_1c_ (%)	Mean (SD)	6.4 (1.2)	5.9 (0.9)	7.1 (1.3)	
	Missing	N (%)	481 (26)	432 (34)	49 (9)	
Baseline SBP	SBP (mm Hg)	Mean (SD)	133 (17)	133 (17)	135 (17)	0.10
	Missing	N (%)	141 (8)	4 (0)	137 (24)	
Baseline total cholesterol	Total cholesterol (mmol/l)	Mean (SD)	5.1 (1.1)	5.3 (1.1)	4.7 (1.1)	**<0.01**
	Missing	N (%)	610 (33)	425 (34)	185 (32)	

P values from Welch two sample t-test for continuous variables, and a z-test for proportions.

P values in bold represent significance at the 5% level.

BMI, body mass index; GLoW, Glucose Lowering through Weight management; HbA1c, glycated haemoglobin; IMD, Index of Multiple Deprivation; NDH, non-diabetic hyperglycaemia; SBP, systolic blood pressure; WRAP, Weight loss Referral for Adults in Primary care.

All risk factors declined on average between baseline and 12 months. Mean weight change was significantly lower in individuals with T2D (−1.24 kg, SD 2.5) than those without T2D (NDN −2.2 kg, SD 2.78; NDH −2.2 kg, SD 2.8). Those with NDH had the largest average reduction in HbA_1c_ (−2.4 mmol/mol, SD 3.1), compared with the decline in those with normoglycaemia (−0.8 mmol/mol, SD 2.4) or T2D (−0.3 mmol/mol, SD 13.0). The change in SBP did not vary between subgroups (mean −3.47 mm Hg, SD 15.33). Total cholesterol change reduced by −0.27 (SD 0.73) mmol/L on average, with a smaller change seen in those with T2D (−0.18 mmol/L, SD 0.81), than those without T2D (NDN −0.33 mmol/L, SD 0.65; NDH –0.34 mmol/L, SD 0.71). Summary statistics across time points and statistical tests of subgroup comparisons are reported in online supplemental table S2.

### Regression results

Changes in BMI and HbA_1c_ are positively related in all subgroups (table 2). The largest relationship is in those with T2D, with a 1 kg/m^2^ change in BMI relating to a 1.5 mmol/mol change in HbA_1c_, compared with a smaller change of 0.6 mmol/mol in those with NDH and an even smaller change of 0.3 mmol/mol in the population with normoglycaemia. Full regression outputs are reported in online supplemental tables S6 and S7. Welch t-tests suggest the relationships are statistically different between all subgroups.

**Figure 1:**
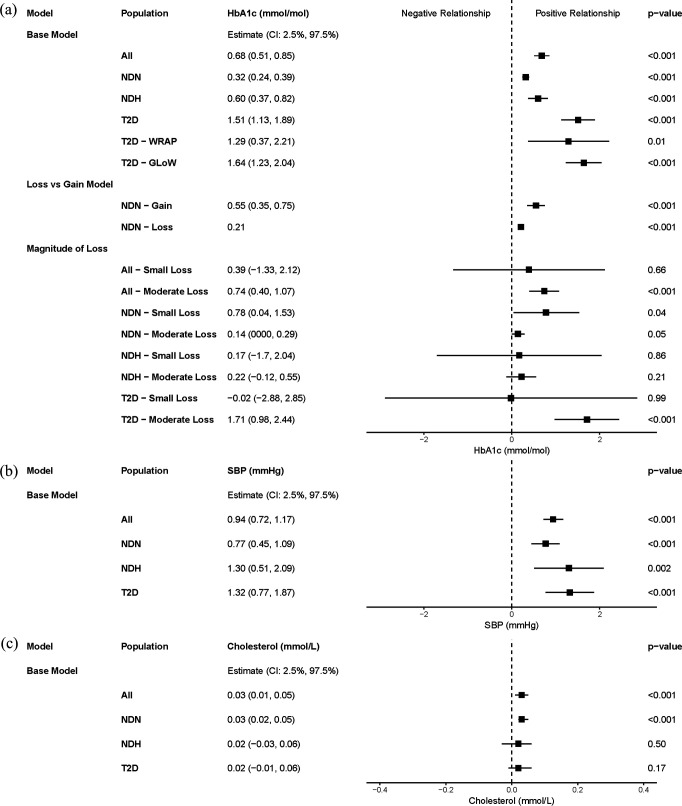
Forest plot of relationships between BMI change and metabolic measures. Coefficients of interest (β_1_) are presented for each regression model with 95% CIs. Regressions were completed by subgroups: non-diabetic normoglycaemia (NDN), non-diabetic hyperglycaemia (NDH), type 2 diabetes (T2D) and all represents the entire sample. (**a**) Coefficients associating BMI and HbA_1c_ (mmol/mol), (**b**) coefficients associating BMI and systolic blood pressure (SBP, mm Hg) and (**c**) coefficients associating BMI and total cholesterol (mmol/L). The loss model coefficient presented for BMI loss is estimated by combining the coefficients for BMI change in weight gain and BMI change in weight loss. BMI, body mass index; GLoW, Glucose Lowering through Weight management; HbA_1c_, glycated haemoglobin; T2D, type 2 diabetes; WRAP, Weight loss Referral for Adults in Primary care.

BMI change is positively related to SBP change and cholesterol change in the pooled population. A 1 kg/m^2^ change in BMI is related to a 0.95 mm Hg change in SBP and a 0.03 mmol/L change in cholesterol. However, diabetes status did not impact these relationships (table 2). Full regression outputs are reported in online supplemental tables S8 and S9. Figure 1 illustrates the differences in the relationships between BMI and HbA_1c_, SBP, and cholesterol across subgroups with CIs.

**Table 2:** Regression outputs associating a change in BMI to a change in HbA_1c_, SBP and cholesterol for the pooled population and for each subgroup: normoglycaemia, non-diabetic hyperglycaemia and T2D

Regression model		n	Risk factor change related to a 1 kg/m^2^ change in BMI				Welch t-test	
Metabolic measure	Population		Risk factor change	SE	95% CI	P value	Against NDH	Against T2D
HbA_1c_ (mmol/mol)	All	1416	0.68	0.09	0.51 to 0.84	**<0.001**		
	NDN	564	0.32	0.04	0.24 to 0.39	**<0.001**	**0.02**	**<0.01**
	NDH	143	0.60	0.11	0.37 to 0.82	**<0.001**		**<0.01**
	T2D	709	1.51	0.20	1.12 to 1.90	**<0.001**	**<0.01**	
SBP (mm Hg)	All	1726	0.95	0.11	0.72 to 1.17	**<0.001**		
	NDN	643	0.77	0.16	0.45 to 1.09	**<0.001**	0.22	0.09
	NDH	160	1.30	0.40	0.51 to 2.10	**0.002**		0.97
	T2D	534	1.33	0.28	0.78 to 1.88	**<0.001**	0.97	
Cholesterol (mmol/L)	All	1106	0.03	0.01	0.01 to 0.05	**<0.001**		
	NDN	554	0.04	0.01	0.02 to 0.05	**<0.001**	0.66	0.26
	NDH	143	0.02	0.02	−0.03 to 0.06	0.504		0.34
	T2D	403	0.02	0.02	−0.01 to 0.06	0.169	0.34	

P values from Welch two sample t-test compare coefficients between models by subgroup.

P values in bold represent significance at the 5% level.

BMI, body mass index; HbA_1c_, glycated haemoglobin; n, observations; NDH, non-diabetic hyperglycaemia; NDN, non-diabetes normoglycaemia; SBP, systolic blood pressure; T2D, type 2 diabetes.

### Additional analyses

**Figure 2:**
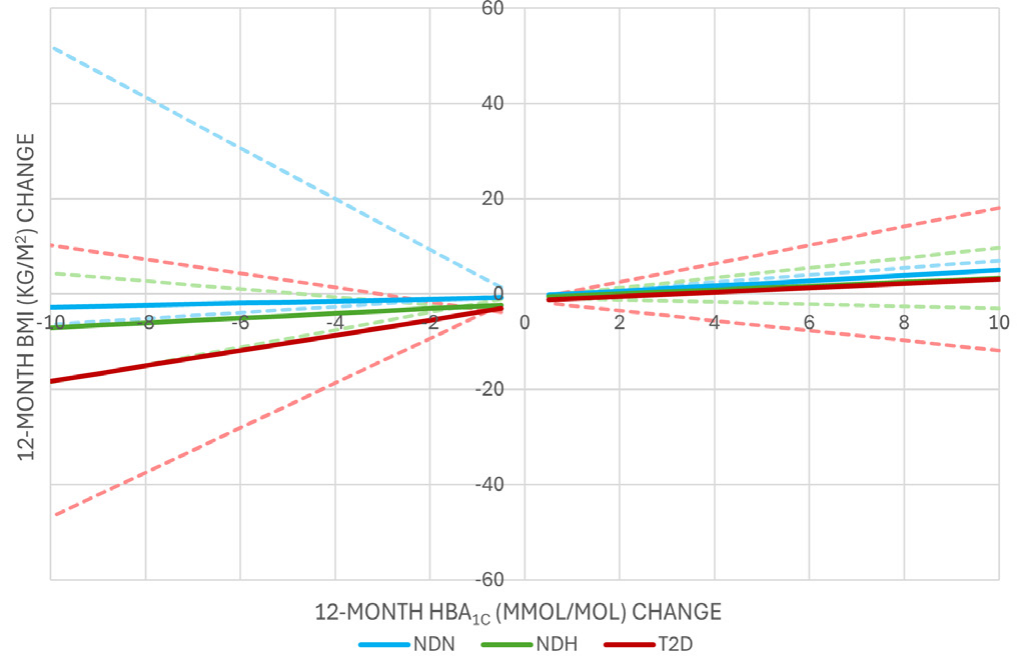
Estimated 12-month HbA1c changes in relation to BMI changes. Resulting HbA_1c_ change was estimated for a white woman, with baseline BMI of 35 kg/m^2^, average baseline age and average HbA_1c_ of each subgroup at each BMI change value between −10 kg/m^2^ and 10 kg/m^2^. This was completed for each subgroup: non-diabetic normoglycaemia (NDN), non-diabetic hyperglycaemia (NDH), type 2 diabetes (T2D). Dotted lines represent the HbA_1c_ change applying BMI change coefficients at the 95% CIs. BMI, body mass index; HbA_1c_, glycated haemoglobin.

The relationship between HbA_1c_ and BMI change is smaller when considering weight loss instead of weight gain in individuals with normoglycaemia (online supplemental tables S10 and S11). A 1 kg/m^2^ gain in BMI would result in a larger increase in HbA_1c_ than the equivalent 1 kg/m^2^ loss would reduce HbA_1c_. Conversely, the relationship is larger when considering weight loss, compared with weight gain, in individuals with hyperglycaemia (either diagnosed with T2D or not) but this difference is not statistically significant (figure 2). Figure 1 illustrates the relationship in BMI gain for individuals with normoglycaemia with CIs, and the relationship in BMI loss estimated by combining the coefficients for BMI change in weight gain and BMI change in weight loss.

There was no consistent narrative as to the impact weight loss or weight gain had on the equivalent relationship with cholesterol and SBP (online supplemental tables S12 and S13).

Table 3 reports the association between BMI and HbA_1c_ in individuals who lost weight, by diabetes status. BMI loss is related to a reduction in HbA_1c_ across all subgroups, with a larger relationship in individuals with T2D. However, this changes when considering the magnitude of weight loss. The relationships in the population with NDH were insignificant when dividing by weight loss magnitude. In individuals with T2D, the relationship was large and significant when considering moderate weight loss, but negligible in small weight loss. A 1 kg reduction in weight relates to a 1.7 mmol/mol reduction in HbA_1c_ in individuals with T2D who achieved moderate weight loss. Conversely, the relationship in individuals with normoglycaemia was larger in the population with small weight loss than the population with moderate weight loss (online supplemental tables S14–S17). Figure 1 illustrates the relationships across these subgroups by magnitude of weight loss with CIs.

**Table 3:** Relationships between BMI loss and HbA_1c_ across different magnitudes of BMI loss for the pooled population and for each subgroup: normoglycaemia, non-diabetic hyperglycaemia and T2D

Population	All BMI loss					
	n	HbA_1c_ change	SE	95% CI	P value	
All	964	0.67	0.12	0.43 to 0.91	**<0.001**	
NDN	379	0.22	0.05	0.13 to 0.32	**<0.001**	
NDH	105	0.41	0.13	0.16 to 0.65	**0.002**	
T2D	480	1.49	0.26	0.97 to 2.01	**<0.001**	

Population	Small BMI loss (<5%)					
	n	HbA_1c_ change	SE	95% CI	P value	
All	476	0.39	0.88	−1.34 to 2.12	0.656	
NDN	153	0.78	0.38	0.03 to 1.53	**0.041**	
NDH	54	0.17	0.95	−1.76 to 2.09	0.861	
T2D	269	−0.02	1.36	−2.90 to 2.86	0.989	

Population	Moderate BMI loss (≥5%)					Welch t-test (<5% vs ≥5%)
	n	HbA_1c_ change	SE	95% CI	P value	P value
All	488	0.74	0.17	0.40 to 1.08	**<0.001**	0.80
NDN	226	0.14	0.07	−0.00 to 0.29	0.055	**<0.01**
NDH	51	0.22	0.17	−0.13 to 0.56	0.207	0.98
T2D	211	1.71	0.37	0.98 to 2.44	**<0.001**	0.57

HbA_1c_ change coefficients represent the change in HbA_1c_ per 1 kg/m2 change in BMI.

P values from Welch two sample t-test compare coefficients between models by magnitude of weight loss.

P values in bold represent significance at the 5% level.

BMI, body mass index; Coef, coefficient; HbA_1c_, glycated haemoglobin; n, observations; NDH, non-diabetes hyperglycaemia; NDN, non-diabetes normoglycaemia; T2D, type 2 diabetes.

Controlling for medication use did not impact the relationships between BMI change and HbA_1c_, SBP or cholesterol changes (online supplemental table S18).

Dividing the population by BMI classification resulted in regression coefficients between BMI change and HbA_1c_ change that were larger in those with severe obesity compared with those with obesity. However, this difference was not significant (Welch t-test p=0.56). The relationship in those with overweight was insignificant. When considering diabetes status subgroups, sample sizes were limited, and analyses were not performed. Regression outputs are reported in online supplemental table S19.

### Sensitivity analysis

Multiple imputation did not change the results substantially for all analyses, except in cholesterol, in which the relationship with the pooled population became insignificant (online supplemental tables S22–24).

A sensitivity analysis with a pooled dataset and a diabetes status indicator and interaction term suggests the relationship in individuals with NDH is not significantly different to that in individuals with normoglycaemia but confirms that BMI change has a greater relation to HbA_1c_ in individuals with T2D than individuals with normoglycaemia (online supplemental table S21).

Despite the difference in the baseline and 12-month change in HbA_1c_ between the trial populations, the relationships between BMI change and HbA_1c_ change were not significantly different between the WRAP and GLoW subgroups for individuals with T2D (Welch test; p=0.497, online supplemental table S20).

Unadjusted estimates for the primary analysis are presented in online supplemental tables S3–S5.

## Discussion

Results suggest BMI change and HbA_1c_ change are positively related, and that the relationship is not stable across subpopulations defined by glycaemic status. The relationship is significantly larger in individuals with T2D than in individuals without T2D. Additionally, there is some evidence that BMI change in individuals with NDH is associated with a larger change in HbA_1c_ compared with individuals with normoglycaemia. However, the difference was not statistically significant in a sensitivity analysis.

The results find that SBP significantly changes with BMI change, but diabetes status does not impact this relationship. BMI change was associated with a change in total cholesterol in the pooled sample; however, there were mixed results across the subpopulations, and these differences were not statistically significant.

Medication use did not change the relationships between BMI change and metabolic outcomes.

Weight gain has a significantly larger impact on HbA_1c_ than weight loss in individuals with normoglycaemia, implying an asymmetric relationship. This asymmetry was not observed for those with NDH or T2D. Individuals without T2D can only exhibit a limited fall in HbA,_1c_ rationalising the flatter relationship with weight loss in this population. However, the relationship remained significant and negative, suggesting that weight loss can lead to reductions in HbA_1c_ in individuals without elevated HbA_1c_. Although the extent to which this negative relationship applied at lower levels of baseline HbA_1c_ was not investigated further within this population.

Only weight loss greater than 5% significantly reduces HbA_1c_ in individuals with T2D. While the 5% threshold was not chosen to reflect a particular turning point of impact, it demonstrates there may be a cut-off, before which weight loss has a limited impact on glucose control. This threshold did not apply in individuals with normoglycaemia, who benefited from marginal reductions in HbA_1c_ in small weight loss.

There is some evidence that the relationship between weight change and HbA_1c_ may differ depending on BMI classification. The relationship was larger in those with severe obesity compared with those with obesity, and it was smaller and insignificant in those overweight. However, the differences were statistically insignificant. Further investigation is required for more conclusive insight.

### Comparison to previous research

A similar analysis of weight loss trial populations found a 1 kg change in weight was related to a 0.1 mmol/mol change in HbA_1c_ for individuals without T2D and a 0.6 mmol/mol change for those with T2D.^1^ Converting the relationships from our analysis to a 1 kg weight change, using the average height in the GLoW trial (1.7 m), found a similar HbA_1c_ change of 0.1 mmol/mol in individuals with normoglycaemia, and 0.5 mmol/mol in individuals with T2D. Similarly, a 1 kg change in weight relates to an average change of 0.3 mm Hg in SBP and 0.01 mmol/L in total cholesterol in the whole population, compared with 0.4 mm Hg and 0.02 mmol/L changes estimated previously.^1^ While there is significant overlap in the non-diabetic population, given the use of the WRAP trial data in both analyses, our analysis sought to contribute by exploring whether heterogeneity within the non-diabetic population could be explained by glycaemic status.

A meta-analysis of RCTs reports a similar conclusion that HbA_1c_ does not change significantly with weight loss of less than 5% of baseline weight, yet does in larger weight losses for individuals with T2D.^6^ Our analysis contributed by demonstrating how this may not apply in individuals with normoglycaemia as a significant relationship only existed in small weight loss. This could be explained by the lack of excess HbA_1c_ in these individuals.

Finally, a meta-analysis of weight loss trials identified a stronger relationship between weight loss and HbA_1c_ in individuals with T2D.^4^ The linear mixed model generated predicts a larger change in HbA_1c_, of −0.40%, compared with −0.22% predicted within our analysis, given an average weight change of −3.5 kg.^4^ The difference could be explained by the types of interventions evaluated in the meta-analysis as they differed from the commercial behavioural weight loss interventions in the WRAP and GLoW trials, including stricter diet control and pharmaceutical interventions.

### Strengths and limitations

Including the GLoW trial data increased the sample size of individuals with T2D. However, generalisability is limited by the demographic composition of the trials, both containing a high proportion of women and white individuals. Although trial population differences were accommodated for through control variables within the regression analysis.

Additional population characteristics may impact the relationships estimated. Smoking cessation and menopause have both previously been observed to relate to changes in weight and cardiometabolic risk; however, data availability meant exploration of this were not possible within this analysis.

While HbA_1c_, blood pressure and cholesterol levels are important predictors of CVD, weight change has been observed to relate to improvements in other metabolic risk factors such as low-density lipoproteins, triglycerides or inflammatory markers.^9 25^ Future research could aim to estimate the relationship between BMI change and these other factors across diabetes status subgroups.

The trials evaluated group-based behavioural weight loss interventions. This limits the applicability of the relationship in the context of alternative interventions such as diet restrictions, physical activity, medication or surgery, which can influence metabolic risk factors differently.^6 20 26^ Further analysis into how these relationships differ between weight loss intervention types, considering T2D status, would be valuable to inform approaches to weight loss in the three subpopulations considered here.

Certain pharmacological treatments for hypertension and hypercholesterolaemia have been associated with reduced glycaemic control and new onset of T2D.^17 18 27^ T2D is associated with reduced control of blood pressure and cholesterol levels.^15 16^ Developments in pharmacological treatments for diabetes and excess weight, such as Semaglutide,^28 29^ may also affect how BMI and HbA_1c_ interact. Further research as to whether these medications change the relationship between BMI and cardiometabolic risk factors may provide important insight.

### Implications of results

Within individuals with overweight or obesity, the National Institute for Health and Care Excellence guidelines recommend a target weight loss of 5%–10% for individuals with T2D and 10% for individuals with NDH.^2 11^ Findings from this analysis are in line with these recommendations. Individuals with T2D were found to have significant metabolic change in weight loss larger than 5% of baseline weight. Assuming an average height and weight of 1.7 m and 98 kg, a 10% wt reduction would relate to a clinically meaningful decline in HbA_1c_ of 5.3 mmol/mol in individuals with T2D. Likewise, a 10% wt decline in the NDH population related to a 2.1 mmol/mol decline in HbA_1c_. Given an average HbA_1c_ of 43.5 mmol/mol in this population, this would reduce average HbA_1c_ below the 42 mmol/mol hyperglycaemia threshold.

The weak relationship between BMI change and cholesterol change would imply a 27% wt loss is required to decrease average cholesterol levels to a healthy range in the normoglycaemia population. Consequently, an alternative weight loss method may change cholesterol levels more effectively.

This analysis highlights the value of weight loss and weight management services in individuals with NDH. The results presented have important applications to health economic modelling by quantifying the cardiometabolic benefits of weight loss. The analyses provide relationships that represent differing metabolic responses to weight loss dependent on diabetes and hyperglycaemia status, which may be relevant when performing economic evaluations. It may inform trial design by indicating a benefit of recruiting participants with NDH separately from individuals with normoglycaemia when investigating weight loss and glycaemia.

## Conclusions

BMI change and HbA_1c_ change are positively related. This relationship increases in magnitude as the level of hyperglycaemia increases. Specifically, individuals with NDH were estimated to have a larger relationship than those with normoglycaemia, and smaller than in those with T2D. This relationship varies with the direction of weight change in individuals with normoglycaemia and is only significant for more substantial weight loss in individuals with T2D.

## Supplementary material

10.1136/bmjopen-2024-095046 online supplemental file 1

## Data Availability

Data are available on reasonable request.
