# Supplementary material for: Effects of weight loss and weight gain on HbA1c, systolic blood pressure and total cholesterol in three subgroups defined by blood glucose: a pooled analysis of two behavioural weight management trials in England
Source: BMJ Open. 2025 Apr 15;15(4):e095046. doi: 10.1136/bmjopen-2024-095046 (PMC12004498; doi:10.1136/bmjopen-2024-095046)
Supplement: online supplemental file 1 [file bmjopen-15-4-s001.docx]

**Supplementary Material**

**Table S1: Baseline statistics by subgroups based on type 2 diabetes status: normoglycaemia, non-diabetic hyperglycaemia and type 2 diabetes.**

|  | |  | **NDN**  **N=564** | **NDH**  **N=136** | **T2D**  **N=767** |
| --- | --- | --- | --- | --- | --- |
| **Age** | **Baseline Age (Years)** | Mean (SD) | 51 (13) | 59 (12) | 60 (12) |
|  | **Missing** | N (%) | 0 (0%) | 0(0%) | 0(0%) |
|  |  |  |  |  |  |
| **Sex** | **Women** | N (%) | 395 (70%) | 88 (65%) | 406 (53%) |
|  | **Men** | N (%) | 169 (30%) | 48 (35%) | 361 (47%) |
|  | **Missing** | N (%) | 0 (0%) | 0 (0%) | 0 (0%) |
|  |  |  |  |  |  |
| **Ethnicity** | **White** | N (%) | 521 (92%) | 113 (83%) | 639 (83%) |
|  | **Missing** | N (%) | 14 (3%) | 7 (5%) | 7 (1%) |
|  |  |  |  |  |  |
| **Diabetes Status** | **No Type 2 Diabetes** | N (%) | 564 (100%) | 0 (0%) | 0 (0%) |
|  | **Type 2 Diabetes** | N (%) | 0 (0%) | 0 (0%) | 767 (100%) |
|  | **High risk of Type 2 Diabetes** | N (%) | 0 (0%) | 136 (100%) | 0 (0%) |
|  | **Missing** | N (%) | 0 (0%) | 0 (0%) | 0 (0%) |
|  |  |  |  |  |  |
| **IMD Score** | **1 – Most Deprived** | N (%) | 179 (32%) | 46 (34%) | 135 (18%) |
|  | **2** | N (%) | 164 (29%) | 36 (27%) | 119 (16%) |
|  | **3** | N (%) | 109 (19%) | 27 (20%) | 169 (22%) |
|  | **4** | N (%) | 64 (11%) | 11 (8%) | 140 (18%) |
|  | **5 – Least Deprived** | N (%) | 47 (8%) | 16 (12%) | 137 (18%) |
|  | **Missing** | N (%) | 1 (0%) | 0 (0%) | 67 (9%) |
|  |  |  |  |  |  |
| **Baseline BMI** | **BMI (kg/m^2^)** | Mean (SD) | 33.8 (4.6) | 34.7 (5.4) | 35.0 (6.7) |
|  | **Missing** | N (%) | 0 (0%) | 0 (0%) | 2 (0%) |
|  |  |  |  |  |  |
| **Baseline HbA_1c_** | **HbA_1c_ (mmol/mol)** | Mean (SD) | 37 (3) | 44 (2) | 55 (14) |
|  | **HbA_1c_ (%)** | Mean (SD) | 5.5 (0.3) | 6.2 (0.2) | 7.2 (1.3) |
|  | **Missing** | N (%) | 0 (0%) | 0 (0%) | 104 (14%) |
|  |  |  |  |  |  |
| **Baseline SBP** | **SBP (mmHg)** | Mean (SD) | 131 (17) | 137 (17) | 136 (18) |
|  | **Missing** | N (%) | 1 (0%) | 0 (0%) | 138 (18%) |
|  |  |  |  |  |  |
| **Baseline Total Cholesterol** | **Total Cholesterol (mmol/l)** | Mean (SD) | 5.4 (1.1) | 5.5 (1.3) | 4.8 (1.1) |
|  | **Missing** | N (%) | 2 (0%) | 0 (0%) | 238 (31%) |
| NDN = No diabetes normoglycaemia, NDH = No diabetes hyperglycemia and T2D = Type 2 diabetes.  BMI = Body mass index, SBP = Systolic blood pressure. SD = standard deviation, N = population size. | | | | | |

**Table S2: Descriptive statistics of mean change in BMI, HbA1c, Systolic Blood Pressure and Cholesterol over time by pooled population and by subgroup.**

|  | | |  |  | **All**  **N = 1844** | **NDN**  **N = 564** | **NDH**  **N = 136** | **T2D**  **N = 767** |  | **Welch test P-value of Difference in Means** | | |
| --- | --- | --- | --- | --- | --- | --- | --- | --- | --- | --- | --- | --- |
|  |  |  | Time Point (month) | Observations | mean (SD) | mean (SD) | mean (SD) | mean (SD) |  | **NDN vs NDH** | **T2D vs NDH** | **T2D vs NDN** |
|  | |  |  |  |  |  |  |  |  |  |  |  |
| BMI (kg/m2) | | Baseline | 0 | 1842 | 34.6 (5.7) | 33.8 (4.6) | 34.7 (5.4) | 35.0 (6.7) |  | 0.07 | 0.64 | **<0.01** |
|  |  |  |  |  |  |  |  |  |  |  |  |  |
|  |  | Average change from baseline | 6 | 383 | -0.77 (1.77) | - | - | -0.77 (1.77) |  |  |  |  |
|  |  |  | 12 | 1120 | -1.82 (2.76) | -2.19 (2.78) | -2.24 (2.69) | -1.24 (2.46) |  | 0.86 | **<0.01** | **<0.01** |
|  |  |  | 60 | 632 | -0.55 (3.28) | -0.40 (3.31) | -0.36 (3.21) | -1.02 (2.99) |  | 0.92 | 0.17 | 0.09 |
|  | |  |  |  |  |  |  |  |  |  |  |  |
| HbA_1c_ (mmol/  mol) | | Baseline | 0 | 1363 | 46  (13) | 37  (3) | 44  (2) | 55  (14) |  | **<0.01** | **<0.01** | **<0.01** |
|  |  |  |  |  |  |  |  |  |  |  |  |  |
|  |  | Average change from baseline | 6 | 358 | -1.75 (10.00) | - | - | -1.75 (10.00) |  |  |  |  |
|  |  |  | 12 | 875 | -0.71 (9.90) | -0.84 (2.37) | -2.41 (3.10) | -0.34 (13.03) |  | **<0.01** | **<0.01** | 0.42 |
|  |  |  | 60 | 599 | 0.13 (9.35) | -0.02 (3.23) | -0.90 (4.99) | 1.76 (21.39) |  | 0.15 | 0.31 | 0.49 |
|  | |  |  |  |  |  |  |  |  |  |  |  |
| HbA_1c_ (%) | | Baseline | 0 |  | 6.4 (1.2) | 5.5 (0.3) | 6.1 (0.1) | 7.2 (1.3) |  | **<0.01** | **<0.01** | **<0.01** |
|  |  |  |  |  |  |  |  |  |  |  |  |  |
|  |  | Average change from baseline | 6 |  | -0.16 (0.92) | - | - | -0.16 (0.92) |  |  |  |  |
|  |  |  | 12 |  | -0.06 (0.91) | -0.08 (0.22) | -0.22 (0.28) | -0.03 (1.19) |  | **<0.01** | **<0.01** | 0.42 |
|  | |  | 60 |  | 0.01 (0.86) | -0.00 (0.30) | -0.08 (0.46) | 0.16 (1.96) |  | 0.15 | 0.31 | 0.49 |
|  | |  |  |  |  |  |  |  |  |  |  |  |
| SBP (mmHg) | | Baseline | 0 | 1703 | 133 (17) | 131 (17) | 137 (17) | 136 (18) |  | **<0.01** | 0.43 | **<0.01** |
|  |  |  |  |  |  |  |  |  |  |  |  |  |
|  |  | Average change from baseline | 6 | 238 | -1.92 (16.77) | - | - | -1.92 (16.77) |  |  |  |  |
|  |  |  | 12 | 1045 | -3.47 (15.33) | -4.72 (13.29) | -4.53 (16.71) | -2.44 (17.56) |  | 0.92 | 0.30 | 0.06 |
|  |  |  | 60 | 642 | -1.32 (16.39) | -0.10 (14.75) | -4.14 (18.06) | -1.54 (19.09) |  | 0.07 | 0.36 | 0.49 |
|  | |  |  |  |  |  |  |  |  |  |  |  |
| Cholesterol (mmol/L) | | Baseline | 0 | 1234 | 5.1 (1.1) | 5.4 (1.1) | 5.5 (1.3) | 4.8 (1.1) |  | 0.36 | **<0.01** | **<0.01** |
|  |  |  |  |  |  |  |  |  |  |  |  |  |
|  |  | Average change from baseline | 6 | 200 | -0.06 (0.74) | - | - | -0.06 (0.74) |  |  |  |  |
|  |  |  | 12 | 679 | -0.27 (0.73) | -0.33 (0.65) | -0.34 (0.71) | -0.18 (0.81) |  | 0.91 | 0.11 | **0.03** |
|  |  |  | 60 | 565 | -0.30 (0.85) | -0.26 (0.82) | -0.37 (0.88) | -0.38 (0.94) |  | 0.38 | 0.95 | 0.38 |
|  | NDN = No diabetes normoglycaemia, NDH = No diabetes hyperglycemia and T2D = Type 2 diabetes.  BMI = Body mass index, SBP = Systolic blood pressure. SD = standard deviation, N = population size.  p-values from Welch two sample t-test. | | | | | | | | | | | |

| **Table S3: Unadjusted Analysis: Regression Outputs associating a change in BMI change to a change in HbA1c (mmol/mol)** | | | | | | | | | | | | | | | | |
| --- | --- | --- | --- | --- | --- | --- | --- | --- | --- | --- | --- | --- | --- | --- | --- | --- |
|  | **All** | | | | **NDN** | | | | **NDH** | | | | **T2D** | | | |
| *Coeffcient* | *Estimates* | *std. Error* | *CI (95%)* | *p* | *Estimates* | *std. Error* | *CI (95%)* | *p* | *Estimates* | *std. Error* | *CI (95%)* | *p* | *Estimates* | *std.*  *Error* | *CI (95%)* | *p* |
| Intercept | 0.078 | 0.304 | -0.518 – 0.673 | 0.798 | 0.029 | 0.137 | -0.239 – 0.297 | 0.832 | -0.817 | 0.353 | -1.515 – -0.118 | **0.022** | 0.766 | 0.579 | -0.371 – 1.903 | 0.186 |
| BMI Change | 0.755 | 0.084 | 0.589 – 0.920 | **<0.001** | 0.332 | 0.034 | 0.265 – 0.400 | **<0.001** | 0.562 | 0.098 | 0.368 – 0.755 | **<0.001** | 1.647 | 0.205 | 1.246 – 2.049 | **<0.001** |
| **Random Effects** | | | | | | | | | | | | | | | | |
| σ^2^ | 36.12 | | | | 3.88 | | | | 9.16 | | | | 70.56 | | | |
| τ_00_ | 49.25 _id_ | | | | 3.26 _id_ | | | | 4.18 _id_ | | | | 82.13 _id_ | | | |
| ICC | 0.58 | | | | 0.46 | | | | 0.31 | | | | 0.54 | | | |
| N | 906 _id_ | | | | 356 _id_ | | | | 91 _id_ | | | | 459 _id_ | | | |
| Observations | 1433 | | | | 571 | | | | 148 | | | | 714 | | | |
| Marginal R^2^ / Conditional R^2^ | 0.049 / 0.598 | | | | 0.136 / 0.531 | | | | 0.187 / 0.441 | | | | 0.087 / 0.578 | | | |

| **Table S4: Unadjusted Analysis: Regression Outputs associating a change in BMI change to a change in Cholesterol (mmol/l)** | | | | | | | | | | | | | | | | |
| --- | --- | --- | --- | --- | --- | --- | --- | --- | --- | --- | --- | --- | --- | --- | --- | --- |
|  | **All** | | | | **NDN** | | | | **NDH** | | | | **T2D** | | | |
| *Coeffcient* | *Estimates* | *std. Error* | *CI (95%)* | *p* | *Estimates* | *std.*  *Error* | *CI (95%)* | *p* | *Estimates* | *std.*  *Error* | *CI (95%)* | *p* | *Estimates* | *std. Error* | *CI (95%)* | *p* |
| Intercept | -0.225 | 0.028 | -0.280 – -0.170 | **<0.001** | -0.257 | 0.039 | -0.333 – -0.181 | **<0.001** | -0.371 | 0.073 | -0.515 – -0.227 | **<0.001** | -0.126 | 0.049 | -0.221 – -0.030 | **0.010** |
| BMI Change | 0.027 | 0.008 | 0.012 – 0.042 | **0.001** | 0.029 | 0.010 | 0.010 – 0.047 | **0.003** | -0.002 | 0.021 | -0.043 – 0.039 | 0.923 | 0.037 | 0.017 | 0.003 – 0.071 | **0.032** |
| **Random Effects** | | | | | | | | | | | | | | | | |
| σ^2^ | 0.30 | | | | 0.28 | | | | 0.47 | | | | 0.28 | | | |
| τ_00_ | 0.30 _id_ | | | | 0.28 _id_ | | | | 0.13 _id_ | | | | 0.37 _id_ | | | |
| ICC | 0.50 | | | | 0.50 | | | | 0.21 | | | | 0.57 | | | |
| N | 741 _id_ | | | | 352 _id_ | | | | 90 _id_ | | | | 294 _id_ | | | |
| Observations | 1123 | | | | 561 | | | | 148 | | | | 408 | | | |
| Marginal R^2^ / Conditional R^2^ | 0.010 / 0.501 | | | | 0.015 / 0.508 | | | | 0.000 / 0.212 | | | | 0.011 / 0.576 | | | |

| **Table S5: Unadjusted Analysis: Regression Outputs associating a change in BMI change to a change in Systolic Blood Pressure (mmHg)** | | | | | | | | | | | | | | | | |
| --- | --- | --- | --- | --- | --- | --- | --- | --- | --- | --- | --- | --- | --- | --- | --- | --- |
|  | **All** | | | | **NDN** | | | | **NDH** | | | | **T2D** | | | |
| *Coeffcient* | *Estimates* | *std. Error* | *CI (95%)* | *p* | *Estimates* | *std. Error* | *CI (95%)* | *p* | *Estimates* | *std. Error* | *CI (95%)* | *p* | *Estimates* | *std. Error* | *CI (95%)* | *p* |
| Intercept | -0.988 | 0.443 | -1.856 – -0.119 | **0.026** | -1.101 | 0.655 | -2.387 – 0.186 | 0.093 | -2.894 | 1.619 | -6.091 – 0.303 | 0.076 | -0.272 | 0.871 | -1.983 – 1.438 | 0.754 |
| BMI Change | 1.213 | 0.122 | 0.974 – 1.452 | **<0.001** | 1.092 | 0.170 | 0.757 – 1.427 | **<0.001** | 1.195 | 0.427 | 0.352 – 2.038 | **0.006** | 1.711 | 0.313 | 1.096 – 2.326 | **<0.001** |
| **Random Effects** | | | | | | | | | | | | | | | | |
| σ^2^ | 128.24 | | | | 116.98 | | | | 171.29 | | | | 122.06 | | | |
| τ_00_ | 105.36 _id_ | | | | 73.29 _id_ | | | | 115.67 _id_ | | | | 155.53 _id_ | | | |
| ICC | 0.45 | | | | 0.39 | | | | 0.40 | | | | 0.56 | | | |
| N | 1127 _id_ | | | | 390 _id_ | | | | 97 _id_ | | | | 377 _id_ | | | |
| Observations | 1768 | | | | 652 | | | | 166 | | | | 543 | | | |
| Marginal R^2^ / Conditional R^2^ | 0.052 / 0.479 | | | | 0.059 / 0.421 | | | | 0.045 / 0.430 | | | | 0.051 / 0.583 | | | |

**Table S6: Base Analysis: Regression Outputs associating a change in BMI change to a change in HbA1c (mmol/mol)**

|  | **All** | | | | **NDN** | | | | **NDH** | | | | | | **T2D** | | | |
| --- | --- | --- | --- | --- | --- | --- | --- | --- | --- | --- | --- | --- | --- | --- | --- | --- | --- | --- |
| *Coefficient* | *Estimates* | *std. Error* | *CI (95%)* | *p* | *Estimates* | *std. Error* | *CI (95%)* | *p* | | *Estimates* | *std. Error* | *CI (95%)* | *p* | *Estimates* | | *std. Error* | *CI (95%)* | *p* |
| Intercept | 4.151 | 2.436 | -0.629 – 8.930 | 0.089 | 5.422 | 1.896 | 1.698 – 9.146 | **0.004** | | -0.566 | 9.891 | -20.130 – 18.997 | 0.954 | 15.627 | | 5.347 | 5.129 – 26.125 | **0.004** |
| BMI Change | 0.680 | 0.086 | 0.510 – 0.849 | **<0.001** | 0.315 | 0.037 | 0.242 – 0.389 | **<0.001** | | 0.597 | 0.114 | 0.371 – 0.823 | **<0.001** | 1.507 | | 0.195 | 1.125 – 1.890 | **<0.001** |
| Baseline Age | 0.006 | 0.022 | -0.037 – 0.048 | 0.801 | 0.018 | 0.011 | -0.004 – 0.039 | 0.105 | | 0.034 | 0.036 | -0.037 – 0.106 | 0.346 | -0.067 | | 0.042 | -0.148 – 0.015 | 0.109 |
| Gender (Female) | -0.102 | 0.554 | -1.188 – 0.984 | 0.854 | -0.643 | 0.274 | -1.182 – -0.104 | **0.019** | | -1.428 | 0.730 | -2.871 – 0.015 | 0.052 | 0.814 | | 0.981 | -1.112 – 2.739 | 0.407 |
| Ethnicity (White) | 0.269 | 0.937 | -1.570 – 2.108 | 0.774 | -0.308 | 0.642 | -1.569 – 0.953 | 0.631 | | 0.362 | 1.138 | -1.889 – 2.613 | 0.751 | 1.569 | | 1.470 | -1.317 – 4.455 | 0.286 |
| Baseline BMI | 0.077 | 0.049 | -0.019 – 0.174 | 0.117 | 0.049 | 0.028 | -0.006 – 0.104 | 0.084 | | 0.192 | 0.068 | 0.057 – 0.326 | **0.005** | 0.098 | | 0.081 | -0.060 – 0.256 | 0.223 |
| Baseline HbA1c | -0.273 | 0.025 | -0.323 – -0.224 | **<0.001** | -0.199 | 0.045 | -0.288 – -0.109 | **<0.001** | | -0.198 | 0.225 | -0.643 – 0.247 | 0.380 | -0.362 | | 0.039 | -0.439 – -0.285 | **<0.001** |
| Trial | 3.891 | 0.720 | 2.479 – 5.302 | **<0.001** |  |  |  |  | |  |  |  |  | 1.888 | | 1.424 | -0.907 – 4.684 | 0.185 |
| Time Point (60 months) | 0.864 | 0.458 | -0.034 – 1.763 | 0.059 | 0.192 | 0.190 | -0.182 – 0.566 | 0.313 | | 0.724 | 0.566 | -0.395 – 1.842 | 0.203 | 6.619 | | 1.405 | 3.861 – 9.377 | **<0.001** |
| Time Point (6 months) | -1.647 | 0.550 | -2.726 – -0.568 | **0.003** |  |  |  |  | |  |  |  |  | -1.708 | | 0.736 | -3.153 – -0.263 | **0.021** |
| **Random Effects** | | | | | | | | | | | | | | | | | | |
| σ^2^ | 35.57 | | | | 3.90 | | | | | 9.35 | | | | 64.15 | | | | |
| τ_00_ | 37.76 _id_ | | | | 2.93 _id_ | | | | | 3.51 _id_ | | | | 60.00 _id_ | | | | |
| ICC | 0.51 | | | | 0.43 | | | | | 0.27 | | | | 0.48 | | | | |
| N | 894 _id_ | | | | 350 _id_ | | | | | 88 _id_ | | | | 456 _id_ | | | | |
| Observations | 1416 | | | | 564 | | | | | 143 | | | | 709 | | | | |
| Marginal R^2^ / Conditional R^2^ | 0.148 / 0.587 | | | | 0.184 / 0.534 | | | | | 0.247 / 0.453 | | | | 0.227 / 0.601 | | | | |

**Table S7: Base Analysis: Regression Outputs associating a change in BMI change to a change in HbA1c (%)**

|  | **All** | | | | **NDN** | | | | **NDH** | | | | **T2D** | | | |
| --- | --- | --- | --- | --- | --- | --- | --- | --- | --- | --- | --- | --- | --- | --- | --- | --- |
| *Coeffcient* | *Estimates* | *std. Error* | *CI (95%)* | *p* | *Estimates* | *std. Error* | *CI (95%)* | *p* | *Estimates* | *std. Error* | *CI (95%)* | *p* | *Estimates* | *std. Error* | *CI (95%)* | *p* |
| Intercept | 0.968 | 0.240 | 0.496 – 1.439 | **<0.001** | 0.923 | 0.256 | 0.421 – 1.425 | **<0.001** | 0.374 | 1.371 | -2.337 – 3.085 | 0.785 | 2.208 | 0.530 | 1.167 – 3.249 | **<0.001** |
| BMI Change | 0.062 | 0.008 | 0.047 – 0.078 | **<0.001** | 0.029 | 0.003 | 0.022 – 0.036 | **<0.001** | 0.055 | 0.010 | 0.034 – 0.075 | **<0.001** | 0.138 | 0.018 | 0.103 – 0.173 | **<0.001** |
| Baseline Age | 0.001 | 0.002 | -0.003 – 0.004 | 0.801 | 0.002 | 0.001 | -0.000 – 0.004 | 0.105 | 0.003 | 0.003 | -0.003 – 0.010 | 0.346 | -0.006 | 0.004 | -0.014 – 0.001 | 0.109 |
| Gender (Female) | -0.009 | 0.051 | -0.109 – 0.090 | 0.854 | -0.059 | 0.025 | -0.108 – -0.010 | **0.019** | -0.131 | 0.067 | -0.263 – 0.001 | 0.052 | 0.074 | 0.090 | -0.102 – 0.251 | 0.407 |
| Ethnicity (White) | 0.025 | 0.086 | -0.144 – 0.193 | 0.774 | -0.028 | 0.059 | -0.144 – 0.087 | 0.631 | 0.033 | 0.104 | -0.173 – 0.239 | 0.751 | 0.144 | 0.134 | -0.121 – 0.408 | 0.286 |
| Baseline BMI | 0.007 | 0.004 | -0.002 – 0.016 | 0.117 | 0.004 | 0.003 | -0.001 – 0.009 | 0.084 | 0.018 | 0.006 | 0.005 – 0.030 | **0.005** | 0.009 | 0.007 | -0.005 – 0.023 | 0.223 |
| Baseline HbA1c | -0.273 | 0.025 | -0.323 – -0.224 | **<0.001** | -0.199 | 0.045 | -0.288 – -0.109 | **<0.001** | -0.198 | 0.225 | -0.643 – 0.247 | 0.380 | -0.362 | 0.039 | -0.439 – -0.285 | **<0.001** |
| Trial | 0.356 | 0.066 | 0.227 – 0.485 | **<0.001** |  |  |  |  |  |  |  |  | 0.173 | 0.130 | -0.083 – 0.429 | 0.185 |
| Time Point (60 months) | 0.079 | 0.042 | -0.003 – 0.161 | 0.059 | 0.018 | 0.017 | -0.017 – 0.052 | 0.313 | 0.066 | 0.052 | -0.036 – 0.169 | 0.203 | 0.606 | 0.129 | 0.353 – 0.858 | **<0.001** |
| Time Point (6 months) | -0.151 | 0.050 | -0.249 – -0.052 | **0.003** |  |  |  |  |  |  |  |  | -0.156 | 0.067 | -0.288 – -0.024 | **0.021** |
| **Random Effects** | | | | | | | | | | | | | | | | |
| σ^2^ | 0.30 | | | | 0.03 | | | | 0.08 | | | | 0.54 | | | |
| τ_00_ | 0.32 _id_ | | | | 0.02 _id_ | | | | 0.03 _id_ | | | | 0.50 _id_ | | | |
| ICC | 0.51 | | | | 0.43 | | | | 0.27 | | | | 0.48 | | | |
| N | 894 _id_ | | | | 350 _id_ | | | | 88 _id_ | | | | 456 _id_ | | | |
| Observations | 1416 | | | | 564 | | | | 143 | | | | 709 | | | |
| Marginal R^2^ / Conditional R^2^ | 0.148 / 0.587 | | | | 0.184 / 0.534 | | | | 0.247 / 0.453 | | | | 0.227 / 0.601 | | | |
| HbA1c (mmol/mol) is converted to HbA1c (%) using the conversion: $HbA1c \left( \% \right)={(HbA1c (mmol/mol)}/{10.929)}+2.15$ | | | | | | | | | | | | | | | | |

**Table S8: Base Analysis: Regression Outputs associating a change in BMI change to a change in Cholesterol (mmol/l)**

|  | **All** | | | | **NDN** | | | | **NDH** | | | | **T2D** | | | |
| --- | --- | --- | --- | --- | --- | --- | --- | --- | --- | --- | --- | --- | --- | --- | --- | --- |
| *Coeffcient* | *Estimates* | *std. Error* | *CI (95%)* | *p* | *Estimates* | *std. Error* | *CI (95%)* | *p* | *Estimates* | *std. Error* | *CI (95%)* | *p* | *Estimates* | *std. Error* | *CI (95%)* | *p* |
| Intercept | 1.227 | 0.256 | 0.724 – 1.729 | **<0.001** | 1.194 | 0.333 | 0.540 – 1.848 | **<0.001** | 1.027 | 0.731 | -0.419 – 2.473 | 0.162 | 1.276 | 0.478 | 0.335 – 2.216 | **0.008** |
| BMI Change | 0.030 | 0.008 | 0.015 – 0.045 | **<0.001** | 0.035 | 0.009 | 0.016 – 0.054 | **<0.001** | 0.015 | 0.023 | -0.030 – 0.060 | 0.504 | 0.023 | 0.017 | -0.010 – 0.057 | 0.169 |
| Baseline Age | -0.002 | 0.002 | -0.006 – 0.002 | 0.357 | 0.001 | 0.003 | -0.004 – 0.006 | 0.675 | 0.003 | 0.007 | -0.011 – 0.017 | 0.646 | -0.005 | 0.004 | -0.012 – 0.002 | 0.135 |
| Gender (Female) | 0.232 | 0.050 | 0.134 – 0.330 | **<0.001** | 0.266 | 0.069 | 0.131 – 0.402 | **<0.001** | 0.063 | 0.162 | -0.258 – 0.384 | 0.698 | 0.218 | 0.085 | 0.052 – 0.384 | **0.010** |
| Ethnicity (White) | 0.166 | 0.099 | -0.028 – 0.359 | 0.093 | 0.175 | 0.165 | -0.150 – 0.500 | 0.292 | 0.052 | 0.233 | -0.410 – 0.514 | 0.823 | 0.081 | 0.159 | -0.231 – 0.393 | 0.610 |
| Baseline BMI | -0.001 | 0.005 | -0.010 – 0.008 | 0.824 | -0.002 | 0.007 | -0.015 – 0.012 | 0.816 | -0.013 | 0.013 | -0.039 – 0.013 | 0.328 | 0.005 | 0.007 | -0.010 – 0.020 | 0.514 |
| Baseline HbA1c | -0.315 | 0.022 | -0.359 – -0.271 | **<0.001** | -0.331 | 0.030 | -0.390 – -0.272 | **<0.001** | -0.214 | 0.067 | -0.347 – -0.080 | **0.002** | -0.332 | 0.041 | -0.412 – -0.253 | **<0.001** |
| Trial | 0.030 | 0.078 | -0.122 – 0.182 | 0.700 |  |  |  |  |  |  |  |  | 0.095 | 0.110 | -0.120 – 0.311 | 0.385 |
| Time Point (60 months) | -0.043 | 0.041 | -0.124 – 0.037 | 0.292 | -0.019 | 0.050 | -0.117 – 0.079 | 0.701 | -0.093 | 0.116 | -0.321 – 0.136 | 0.425 | -0.079 | 0.097 | -0.270 – 0.111 | 0.414 |
| Time Point (6 months) | 0.037 | 0.077 | -0.115 – 0.189 | 0.630 |  |  |  |  |  |  |  |  | 0.038 | 0.079 | -0.117 – 0.193 | 0.628 |
| **Random Effects** | | | | | | | | | | | | | | | | |
| σ^2^ | 0.29 | | | | 0.27 | | | | 0.40 | | | | 0.29 | | | |
| τ_00_ | 0.19 _id_ | | | | 0.16 _id_ | | | | 0.13 _id_ | | | | 0.24 _id_ | | | |
| ICC | 0.39 | | | | 0.37 | | | | 0.24 | | | | 0.46 | | | |
| N | 729 _id_ | | | | 346 _id_ | | | | 87 _id_ | | | | 291 _id_ | | | |
| Observations | 1106 | | | | 554 | | | | 143 | | | | 403 | | | |
| Marginal R^2^ / Conditional R^2^ | 0.206 / 0.516 | | | | 0.236 / 0.522 | | | | 0.104 / 0.319 | | | | 0.200 / 0.567 | | | |

**Table S9: Base Analysis: Regression Outputs associating a change in BMI change to a change in Systolic Blood Pressure (mmHg)**

|  | **All** | | | | | | | **NDN** | | | | | | | | **NDH** | | | | | | | | **T2D** | | | | | | | | |
| --- | --- | --- | --- | --- | --- | --- | --- | --- | --- | --- | --- | --- | --- | --- | --- | --- | --- | --- | --- | --- | --- | --- | --- | --- | --- | --- | --- | --- | --- | --- | --- | --- |
| *Coeffcient* | *Estimates* | *std. Error* | | *CI (95%)* | | *p* | | *Estimates* | | *std. Error* | | *CI (95%)* | | *p* | | *Estimates* | | *std. Error* | | *CI (95%)* | | *p* | | *Estimates* | | *std. Error* | | *CI (95%)* | | *p* | | |
| Intercept | 48.048 | | 3.800 | | 40.595 – 55.501 | | **<0.001** | | 49.990 | | 5.807 | | 38.586 – 61.393 | | **<0.001** | | 64.683 | | 12.440 | | 40.103 – 89.264 | | **<0.001** | | 61.682 | | 7.446 | | 47.053 – 76.311 | | **<0.001** |  |
| BMI Change | 0.945 | | 0.114 | | 0.721 – 1.169 | | **<0.001** | | 0.767 | | 0.164 | | 0.445 – 1.089 | | **<0.001** | | 1.301 | | 0.403 | | 0.505 – 2.097 | | **0.002** | | 1.319 | | 0.279 | | 0.770 – 1.868 | | **<0.001** |  |
| Baseline Age | 0.099 | | 0.029 | | 0.042 – 0.156 | | **0.001** | | 0.080 | | 0.047 | | -0.012 – 0.172 | | 0.089 | | 0.045 | | 0.123 | | -0.198 – 0.288 | | 0.716 | | 0.024 | | 0.057 | | -0.088 – 0.135 | | 0.678 |  |
| Gender (Female) | -2.043 | | 0.723 | | -3.461 – -0.624 | | **0.005** | | -1.746 | | 1.155 | | -4.013 – 0.521 | | 0.131 | | -3.211 | | 2.505 | | -8.160 – 1.737 | | 0.202 | | -2.021 | | 1.321 | | -4.615 – 0.573 | | 0.127 |  |
| Ethnicity (White) | 0.894 | | 1.383 | | -1.819 – 3.607 | | 0.518 | | -2.154 | | 2.598 | | -7.256 – 2.947 | | 0.407 | | 2.728 | | 3.828 | | -4.836 – 10.293 | | 0.477 | | 4.594 | | 2.382 | | -0.084 – 9.273 | | 0.054 |  |
| Baseline BMI | 0.189 | | 0.068 | | 0.055 – 0.323 | | **0.006** | | -0.000 | | 0.116 | | -0.229 – 0.228 | | 0.998 | | 0.412 | | 0.233 | | -0.050 – 0.873 | | 0.080 | | 0.166 | | 0.116 | | -0.061 – 0.393 | | 0.152 |  |
| Baseline HbA1c | -0.473 | | 0.020 | | -0.513 – -0.433 | | **<0.001** | | -0.407 | | 0.033 | | -0.472 – -0.342 | | **<0.001** | | -0.603 | | 0.068 | | -0.738 – -0.468 | | **<0.001** | | -0.537 | | 0.036 | | -0.608 – -0.466 | | **<0.001** |  |
| Trial | 2.088 | | 1.289 | | -0.439 – 4.615 | | 0.105 | |  | |  | |  | |  | |  | |  | |  | |  | | 0.682 | | 1.770 | | -2.796 – 4.159 | | 0.700 |  |
| Time Point (60 months) | 0.958 | | 0.654 | | -0.324 – 2.240 | | 0.143 | | 2.900 | | 0.923 | | 1.087 – 4.713 | | **0.002** | | -3.125 | | 2.323 | | -7.714 – 1.465 | | 0.181 | | 0.431 | | 1.599 | | -2.710 – 3.572 | | 0.788 |  |
| Time Point (6 months) | -0.855 | | 1.399 | | -3.598 – 1.888 | | 0.541 | |  | |  | |  | |  | |  | |  | |  | |  | | -0.762 | | 1.396 | | -3.504 – 1.981 | | 0.585 |  |
| **Random Effects** | | | | | | | | | | | | | | | | | | | | | | | | | | | | | | | | |
| σ^2^ | 129.94 | | | | | | | 117.37 | | | | | | | | 192.94 | | | | | | | | 120.60 | | | | | | | | |
| τ_00_ | 38.31 _id_ | | | | | | | 28.18 _id_ | | | | | | | | 2.95 _id_ | | | | | | | | 65.18 _id_ | | | | | | | | |
| ICC | 0.23 | | | | | | | 0.19 | | | | | | | | 0.02 | | | | | | | | 0.35 | | | | | | | | |
| N | 1099 _id_ | | | | | | | 383 _id_ | | | | | | | | 94 _id_ | | | | | | | | 372 _id_ | | | | | | | | |
| Observations | 1726 | | | | | | | 643 | | | | | | | | 160 | | | | | | | | 534 | | | | | | | | |
| Marginal R^2^ / Conditional R^2^ | 0.314 / 0.470 | | | | | | | 0.285 / 0.423 | | | | | | | | 0.387 / 0.396 | | | | | | | | 0.384 / 0.600 | | | | | | | | |

|  | **All** | | | | **NDN** | | | | **NDH** | | | | **T2D** | | | |
| --- | --- | --- | --- | --- | --- | --- | --- | --- | --- | --- | --- | --- | --- | --- | --- | --- |
| *Coeffcient* | *Estimates* | *std. Error* | *CI (95%)* | *p* | *Estimates* | *std. Error* | *CI (95%)* | *p* | *Estimates* | *std. Error* | *CI (95%)* | *p* | *Estimates* | *std. Error* | *CI (95%)* | *p* |
| Intercept | 5.021 | 2.478 | 0.161 – 9.881 | **0.043** | 5.446 | 1.894 | 1.725 – 9.167 | **0.004** | 1.414 | 10.224 | -18.811 – 21.640 | 0.890 | 16.826 | 5.445 | 6.136 – 27.516 | **0.002** |
| BMI Change | 0.234 | 0.258 | -0.272 – 0.740 | 0.365 | 0.549 | 0.102 | 0.348 – 0.749 | **<0.001** | 0.407 | 0.321 | -0.229 – 1.043 | 0.208 | 0.449 | 0.762 | -1.047 – 1.945 | 0.556 |
| Baseline Age | 0.004 | 0.022 | -0.040 – 0.047 | 0.867 | 0.022 | 0.011 | 0.001 – 0.044 | **0.043** | 0.036 | 0.037 | -0.038 – 0.110 | 0.334 | -0.070 | 0.042 | -0.152 – 0.012 | 0.093 |
| Gender (Female) | -0.087 | 0.554 | -1.173 – 1.000 | 0.876 | -0.653 | 0.274 | -1.190 – -0.115 | **0.017** | -1.511 | 0.748 | -2.991 – -0.031 | **0.045** | 0.923 | 0.987 | -1.016 – 2.861 | 0.350 |
| Ethnicity (White) | 0.274 | 0.937 | -1.564 – 2.113 | 0.770 | -0.402 | 0.640 | -1.660 – 0.855 | 0.530 | 0.145 | 1.183 | -2.195 – 2.485 | 0.903 | 1.652 | 1.476 | -1.245 – 4.549 | 0.263 |
| Baseline BMI | 0.083 | 0.050 | -0.014 – 0.180 | 0.094 | 0.033 | 0.028 | -0.023 – 0.089 | 0.245 | 0.186 | 0.071 | 0.046 – 0.326 | **0.009** | 0.104 | 0.081 | -0.055 – 0.263 | 0.201 |
| Baseline HbA1c | -0.274 | 0.026 | -0.324 – -0.224 | **<0.001** | -0.194 | 0.045 | -0.283 – -0.105 | **<0.001** | -0.215 | 0.231 | -0.673 – 0.242 | 0.354 | -0.365 | 0.040 | -0.443 – -0.288 | **<0.001** |
| Loss | -0.935 | 0.609 | -2.131 – 0.260 | 0.125 | -0.185 | 0.307 | -0.788 – 0.418 | 0.547 | -1.283 | 0.942 | -3.148 – 0.581 | 0.176 | -0.875 | 1.187 | -3.205 – 1.454 | 0.461 |
| Trial | 3.809 | 0.721 | 2.395 – 5.223 | **<0.001** |  |  |  |  |  |  |  |  | 1.870 | 1.426 | -0.930 – 4.670 | 0.190 |
| Time Point (60 months) | 0.898 | 0.462 | -0.009 – 1.804 | 0.052 | 0.094 | 0.192 | -0.282 – 0.471 | 0.623 | 0.632 | 0.560 | -0.475 – 1.739 | 0.261 | 6.731 | 1.401 | 3.980 – 9.483 | **<0.001** |
| Time Point (6 months) | -1.680 | 0.551 | -2.760 – -0.600 | **0.002** |  |  |  |  |  |  |  |  | -1.813 | 0.738 | -3.262 – -0.364 | **0.014** |
| BMI Change * Loss | 0.459 | 0.287 | -0.104 – 1.021 | 0.110 | -0.342 | 0.115 | -0.568 – -0.115 | **0.003** | 0.094 | 0.353 | -0.604 – 0.792 | 0.790 | 1.154 | 0.813 | -0.443 – 2.750 | 0.156 |
| **Random Effects** | | | | | | | | | | | | | | | | |
| σ^2^ | 35.48 | | | | 3.79 | | | | 8.91 | | | | 63.44 | | | |
| τ_00_ | 37.80 _id_ | | | | 2.96 _id_ | | | | 4.14 _id_ | | | | 61.13 _id_ | | | |
| ICC | 0.52 | | | | 0.44 | | | | 0.32 | | | | 0.49 | | | |
| N | 894 _id_ | | | | 350 _id_ | | | | 88 _id_ | | | | 456 _id_ | | | |
| Observations | 1416 | | | | 564 | | | | 143 | | | | 709 | | | |
| Marginal R^2^ / Conditional R^2^ | 0.150 / 0.589 | | | | 0.195 / 0.548 | | | | 0.252 / 0.489 | | | | 0.228 / 0.607 | | | |

**Table S10: BMI Loss versus BMI Gain analysis: Regression outputs of the association between a change in BMI and a change in HbA1c (mmol/mol) with interactions term for weight loss.**

**Table S11: BMI Loss versus BMI Gain analysis: Regression outputs of the association between a change in BMI and a change in HbA1c (%) with interactions term for weight loss.**

|  | **All** | | | | **NDN** | | | | **NDH** | | | | **T2D** | | | |
| --- | --- | --- | --- | --- | --- | --- | --- | --- | --- | --- | --- | --- | --- | --- | --- | --- |
| *Coeffcient* | *Estimates* | *std. Error* | *CI (95%)* | *p* | *Estimates* | *std. Error* | *CI (95%)* | *p* | *Estimates* | *std. Error* | *CI (95%)* | *p* | *Estimates* | *std. Error* | *CI (95%)* | *p* |
| Intercept | 1.048 | 0.244 | 0.570 – 1.526 | **<0.001** | 0.915 | 0.255 | 0.414 – 1.416 | **<0.001** | 0.592 | 1.413 | -2.204 – 3.388 | 0.676 | 2.325 | 0.539 | 1.267 – 3.383 | **<0.001** |
| BMI Change | 0.021 | 0.024 | -0.025 – 0.068 | 0.365 | 0.050 | 0.009 | 0.032 – 0.069 | **<0.001** | 0.037 | 0.029 | -0.021 – 0.095 | 0.208 | 0.041 | 0.070 | -0.096 – 0.178 | 0.556 |
| Baseline Age | 0.000 | 0.002 | -0.004 – 0.004 | 0.867 | 0.002 | 0.001 | 0.000 – 0.004 | **0.043** | 0.003 | 0.003 | -0.003 – 0.010 | 0.334 | -0.006 | 0.004 | -0.014 – 0.001 | 0.093 |
| Gender (Female) | -0.008 | 0.051 | -0.107 – 0.091 | 0.876 | -0.060 | 0.025 | -0.109 – -0.011 | **0.017** | -0.138 | 0.068 | -0.274 – -0.003 | **0.045** | 0.084 | 0.090 | -0.093 – 0.262 | 0.350 |
| Ethnicity (White) | 0.025 | 0.086 | -0.143 – 0.193 | 0.770 | -0.037 | 0.059 | -0.152 – 0.078 | 0.530 | 0.013 | 0.108 | -0.201 – 0.227 | 0.903 | 0.151 | 0.135 | -0.114 – 0.416 | 0.263 |
| Baseline BMI | 0.008 | 0.005 | -0.001 – 0.016 | 0.094 | 0.003 | 0.003 | -0.002 – 0.008 | 0.245 | 0.017 | 0.006 | 0.004 – 0.030 | **0.009** | 0.009 | 0.007 | -0.005 – 0.024 | 0.201 |
| Baseline HbA1c | -0.274 | 0.026 | -0.324 – -0.224 | **<0.001** | -0.194 | 0.045 | -0.283 – -0.105 | **<0.001** | -0.215 | 0.231 | -0.673 – 0.242 | 0.354 | -0.365 | 0.040 | -0.443 – -0.288 | **<0.001** |
| Loss | -0.086 | 0.056 | -0.195 – 0.024 | 0.125 | -0.017 | 0.028 | -0.072 – 0.038 | 0.547 | -0.117 | 0.086 | -0.288 – 0.053 | 0.176 | -0.080 | 0.109 | -0.293 – 0.133 | 0.461 |
| Trial | 0.349 | 0.066 | 0.219 – 0.478 | **<0.001** |  |  |  |  |  |  |  |  | 0.171 | 0.130 | -0.085 – 0.427 | 0.190 |
| Time Point (60 months) | 0.082 | 0.042 | -0.001 – 0.165 | 0.052 | 0.009 | 0.018 | -0.026 – 0.043 | 0.623 | 0.058 | 0.051 | -0.043 – 0.159 | 0.261 | 0.616 | 0.128 | 0.364 – 0.868 | **<0.001** |
| Time Point (6 months) | -0.154 | 0.050 | -0.253 – -0.055 | **0.002** |  |  |  |  |  |  |  |  | -0.166 | 0.068 | -0.298 – -0.033 | **0.014** |
| BMI Change * Loss | 0.042 | 0.026 | -0.010 – 0.093 | 0.110 | -0.031 | 0.011 | -0.052 – -0.011 | **0.003** | 0.009 | 0.032 | -0.055 – 0.072 | 0.790 | 0.106 | 0.074 | -0.041 – 0.252 | 0.156 |
| **Random Effects** | | | | | | | | | | | | | | | | |
| σ^2^ | 0.30 | | | | 0.03 | | | | 0.07 | | | | 0.53 | | | |
| τ_00_ | 0.32 _id_ | | | | 0.02 _id_ | | | | 0.03 _id_ | | | | 0.51 _id_ | | | |
| ICC | 0.52 | | | | 0.44 | | | | 0.32 | | | | 0.49 | | | |
| N | 894 _id_ | | | | 350 _id_ | | | | 88 _id_ | | | | 456 _id_ | | | |
| Observations | 1416 | | | | 564 | | | | 143 | | | | 709 | | | |
| Marginal R^2^ / Conditional R^2^ | 0.150 / 0.589 | | | | 0.195 / 0.548 | | | | 0.252 / 0.489 | | | | 0.228 / 0.607 | | | |

**Table S12: BMI Loss versus BMI Gain analysis: Regression outputs of the association between a change in BMI and a change in Cholesterol (mmol/L) with interactions term for weight loss.**

|  | **All** | | | | **NDN** | | | | **NDH** | | | | **T2D** | | | |
| --- | --- | --- | --- | --- | --- | --- | --- | --- | --- | --- | --- | --- | --- | --- | --- | --- |
| *Coeffcient* | *Estimates* | *std. Error* | *CI (95%)* | *p* | *Estimates* | *std. Error* | *CI (95%)* | *p* | *Estimates* | *std. Error* | *CI (95%)* | *p* | *Estimates* | *std. Error* | *CI (95%)* | *p* |
| Intercept | 1.212 | 0.260 | 0.701 – 1.723 | **<0.001** | 1.128 | 0.336 | 0.469 – 1.788 | **0.001** | 1.206 | 0.736 | -0.249 – 2.661 | 0.104 | 1.132 | 0.492 | 0.166 – 2.099 | **0.022** |
| BMI Change | 0.008 | 0.023 | -0.038 – 0.054 | 0.732 | -0.016 | 0.027 | -0.070 – 0.037 | 0.550 | 0.033 | 0.064 | -0.094 – 0.160 | 0.609 | 0.084 | 0.065 | -0.044 – 0.212 | 0.196 |
| Baseline Age | -0.002 | 0.002 | -0.006 – 0.002 | 0.264 | -0.000 | 0.003 | -0.005 – 0.005 | 0.983 | 0.005 | 0.007 | -0.009 – 0.019 | 0.481 | -0.005 | 0.004 | -0.012 – 0.002 | 0.150 |
| Gender (Female) | 0.239 | 0.050 | 0.140 – 0.337 | **<0.001** | 0.271 | 0.069 | 0.136 – 0.406 | **<0.001** | 0.000 | 0.161 | -0.318 – 0.318 | 1.000 | 0.211 | 0.085 | 0.043 – 0.379 | **0.014** |
| Ethnicity (White) | 0.173 | 0.099 | -0.021 – 0.366 | 0.081 | 0.199 | 0.165 | -0.124 – 0.523 | 0.227 | 0.032 | 0.231 | -0.425 – 0.489 | 0.892 | 0.084 | 0.159 | -0.228 – 0.396 | 0.597 |
| Baseline BMI | 0.000 | 0.005 | -0.009 – 0.009 | 0.995 | 0.002 | 0.007 | -0.012 – 0.016 | 0.787 | -0.019 | 0.013 | -0.046 – 0.007 | 0.150 | 0.005 | 0.008 | -0.010 – 0.020 | 0.499 |
| Baseline Chol | -0.316 | 0.022 | -0.360 – -0.272 | **<0.001** | -0.330 | 0.030 | -0.389 – -0.271 | **<0.001** | -0.187 | 0.067 | -0.319 – -0.055 | **0.006** | -0.326 | 0.041 | -0.407 – -0.246 | **<0.001** |
| Loss | 0.044 | 0.060 | -0.073 – 0.162 | 0.458 | 0.067 | 0.080 | -0.091 – 0.225 | 0.406 | -0.327 | 0.193 | -0.708 – 0.055 | 0.093 | 0.130 | 0.106 | -0.078 – 0.338 | 0.219 |
| Trial | 0.028 | 0.078 | -0.125 – 0.180 | 0.723 |  |  |  |  |  |  |  |  | 0.093 | 0.110 | -0.122 – 0.309 | 0.395 |
| Time Point (60 months) | -0.033 | 0.041 | -0.114 – 0.049 | 0.429 | 0.005 | 0.050 | -0.094 – 0.104 | 0.925 | -0.119 | 0.116 | -0.349 – 0.111 | 0.306 | -0.082 | 0.097 | -0.273 – 0.110 | 0.403 |
| Time Point (6 months) | 0.030 | 0.077 | -0.122 – 0.182 | 0.699 |  |  |  |  |  |  |  |  | 0.043 | 0.080 | -0.114 – 0.199 | 0.591 |
| BMI Change * Loss | 0.035 | 0.026 | -0.016 – 0.086 | 0.178 | 0.077 | 0.030 | 0.017 – 0.137 | **0.012** | -0.067 | 0.070 | -0.206 – 0.072 | 0.344 | -0.054 | 0.069 | -0.190 – 0.082 | 0.438 |
| **Random Effects** | | | | | | | | | | | | | | | | |
| σ^2^ | 0.29 | | | | 0.26 | | | | 0.40 | | | | 0.29 | | | |
| τ_00_ | 0.19 _id_ | | | | 0.16 _id_ | | | | 0.11 _id_ | | | | 0.24 _id_ | | | |
| ICC | 0.39 | | | | 0.37 | | | | 0.21 | | | | 0.46 | | | |
| N | 729 _id_ | | | | 346 _id_ | | | | 87 _id_ | | | | 291 _id_ | | | |
| Observations | 1106 | | | | 554 | | | | 143 | | | | 403 | | | |
| Marginal R^2^ / Conditional R^2^ | 0.208 / 0.516 | | | | 0.246 / 0.528 | | | | 0.134 / 0.315 | | | | 0.202 / 0.568 | | | |

**Table S13: BMI Loss versus BMI Gain analysis: Regression outputs of the association between a change in BMI and a change in Systolic Blood Pressure (mmHg) with interactions term for weight loss.**

|  | **All** | | | | **NDN** | | | | **NDH** | | | | **T2D** | | | |
| --- | --- | --- | --- | --- | --- | --- | --- | --- | --- | --- | --- | --- | --- | --- | --- | --- |
| *Coeffcient* | *Estimates* | *std. Error* | *CI (95%)* | *p* | *Estimates* | *std. Error* | *CI (95%)* | *p* | *Estimates* | *std. Error* | *CI (95%)* | *p* | *Estimates* | *std. Error* | *CI (95%)* | *p* |
| Intercept | 47.358 | 3.874 | 39.761 – 54.955 | **<0.001** | 48.617 | 5.885 | 37.061 – 60.173 | **<0.001** | 65.632 | 12.835 | 40.268 – 90.997 | **<0.001** | 61.762 | 7.606 | 46.820 – 76.704 | **<0.001** |
| BMI Change | 0.980 | 0.347 | 0.300 – 1.660 | **0.005** | 1.287 | 0.438 | 0.428 – 2.146 | **0.003** | 0.801 | 1.236 | -1.642 – 3.243 | 0.518 | 0.602 | 0.932 | -1.230 – 2.433 | 0.519 |
| Baseline Age | 0.096 | 0.029 | 0.039 – 0.153 | **0.001** | 0.076 | 0.047 | -0.017 – 0.169 | 0.108 | 0.040 | 0.124 | -0.205 – 0.286 | 0.745 | 0.021 | 0.057 | -0.091 – 0.133 | 0.712 |
| Gender (Female) | -2.000 | 0.725 | -3.421 – -0.578 | **0.006** | -1.665 | 1.155 | -3.934 – 0.603 | 0.150 | -3.185 | 2.523 | -8.170 – 1.801 | 0.209 | -1.908 | 1.326 | -4.513 – 0.697 | 0.151 |
| Ethnicity (White) | 0.954 | 1.384 | -1.761 – 3.669 | 0.491 | -2.029 | 2.601 | -7.137 – 3.079 | 0.436 | 2.543 | 3.889 | -5.143 – 10.229 | 0.514 | 4.684 | 2.386 | -0.002 – 9.371 | 0.050 |
| Baseline BMI | 0.197 | 0.069 | 0.062 – 0.332 | **0.004** | -0.007 | 0.118 | -0.238 – 0.224 | 0.954 | 0.429 | 0.243 | -0.052 – 0.909 | 0.080 | 0.178 | 0.116 | -0.051 – 0.406 | 0.128 |
| Baseline SBP | -0.474 | 0.020 | -0.514 – -0.434 | **<0.001** | -0.406 | 0.033 | -0.471 – -0.341 | **<0.001** | -0.605 | 0.070 | -0.744 – -0.467 | **<0.001** | -0.538 | 0.036 | -0.608 – -0.467 | **<0.001** |
| Loss | 0.972 | 0.908 | -0.809 – 2.754 | 0.285 | 1.949 | 1.395 | -0.791 – 4.689 | 0.163 | -0.702 | 3.646 | -7.908 – 6.503 | 0.847 | 0.181 | 1.706 | -3.171 – 3.532 | 0.916 |
| Trial | 2.160 | 1.291 | -0.372 – 4.691 | 0.094 |  |  |  |  |  |  |  |  | 0.740 | 1.773 | -2.744 – 4.224 | 0.677 |
| Time Point (60 months) | 1.048 | 0.663 | -0.252 – 2.349 | 0.114 | 2.943 | 0.939 | 1.100 – 4.787 | **0.002** | -3.050 | 2.356 | -7.706 – 1.606 | 0.198 | 0.695 | 1.622 | -2.491 – 3.881 | 0.669 |
| Time Point (6 months) | -0.928 | 1.401 | -3.676 – 1.820 | 0.508 |  |  |  |  |  |  |  |  | -0.952 | 1.410 | -3.722 – 1.819 | 0.500 |
| BMI Change * Loss | 0.082 | 0.383 | -0.669 – 0.834 | 0.830 | -0.460 | 0.499 | -1.440 – 0.520 | 0.357 | 0.577 | 1.347 | -2.086 – 3.240 | 0.669 | 0.960 | 1.020 | -1.043 – 2.963 | 0.347 |
| **Random Effects** | | | | | | | | | | | | | | | | |
| σ^2^ | 129.97 | | | | 117.65 | | | | 195.39 | | | | 121.00 | | | |
| τ_00_ | 38.34 _id_ | | | | 27.73 _id_ | | | | 2.86 _id_ | | | | 65.07 _id_ | | | |
| ICC | 0.23 | | | | 0.19 | | | | 0.01 | | | | 0.35 | | | |
| N | 1099 _id_ | | | | 383 _id_ | | | | 94 _id_ | | | | 372 _id_ | | | |
| Observations | 1726 | | | | 643 | | | | 160 | | | | 534 | | | |
| Marginal R^2^ / Conditional R^2^ | 0.314 / 0.470 | | | | 0.287 / 0.423 | | | | 0.384 / 0.393 | | | | 0.385 / 0.600 | | | |

**Table S14: Moderate Versus Large Weight Loss analysis: Regression outputs of the association between a change in BMI and a change in HbA1c (mmol/mol) in the whole population divided by magnitude of weight loss**

|  | **All Loss** | | | | **BMI loss < 5%** | | | | **BMI loss >= 5%** | | | |
| --- | --- | --- | --- | --- | --- | --- | --- | --- | --- | --- | --- | --- |
| *Coeffcient* | *Estimates* | *std. Error* | *CI (95%)* | *p* | *Estimates* | *std. Error* | *CI (95%)* | *p* | *Estimates* | *std. Error* | *CI (95%)* | *p* |
| Intercept | 6.998 | 3.160 | 0.797 – 13.199 | **0.027** | 2.997 | 4.461 | -5.768 – 11.763 | 0.502 | 9.475 | 3.862 | 1.885 – 17.064 | **0.015** |
| BMI Change | 0.671 | 0.122 | 0.432 – 0.911 | **<0.001** | 0.392 | 0.880 | -1.336 – 2.121 | 0.656 | 0.736 | 0.172 | 0.397 – 1.075 | **<0.001** |
| Baseline Age | 0.010 | 0.028 | -0.046 – 0.065 | 0.730 | -0.014 | 0.039 | -0.090 – 0.062 | 0.718 | 0.033 | 0.035 | -0.035 – 0.101 | 0.342 |
| Gender (Female) | 0.210 | 0.709 | -1.181 – 1.600 | 0.767 | -0.116 | 0.918 | -1.919 – 1.687 | 0.900 | 0.776 | 0.911 | -1.014 – 2.566 | 0.395 |
| Ethnicity (White) | 0.355 | 1.247 | -2.092 – 2.803 | 0.776 | 1.712 | 1.479 | -1.194 – 4.618 | 0.248 | -0.846 | 1.749 | -4.283 – 2.591 | 0.629 |
| Baseline BMI | 1.897 | 0.889 | 0.152 – 3.643 | **0.033** | 3.789 | 1.318 | 1.198 – 6.380 | **0.004** | 0.039 | 1.174 | -2.267 – 2.345 | 0.974 |
| Baseline HbA1c | 0.022 | 0.063 | -0.101 – 0.146 | 0.724 | 0.045 | 0.090 | -0.131 – 0.221 | 0.614 | 0.002 | 0.079 | -0.153 – 0.156 | 0.983 |
| Trial | -0.257 | 0.032 | -0.320 – -0.195 | **<0.001** | -0.238 | 0.042 | -0.320 – -0.156 | **<0.001** | -0.260 | 0.042 | -0.343 – -0.177 | **<0.001** |
| Time Point (60 months) | 1.358 | 0.517 | 0.343 – 2.374 | **0.009** | 1.685 | 1.090 | -0.457 – 3.826 | 0.123 | 1.958 | 0.748 | 0.489 – 3.427 | **0.009** |
| Time Point (6 months) | -0.521 | 0.646 | -1.788 – 0.746 | 0.420 | -1.081 | 1.153 | -3.346 – 1.185 | 0.349 | -1.201 | 0.992 | -3.150 – 0.748 | 0.227 |
| **Random Effects** | | | | | | | | | | | | |
| σ^2^ | 26.49 | | | | 48.88 | | | | 26.24 | | | |
| τ_00_ | 57.27 _id_ | | | | 38.28 _id_ | | | | 44.07 _id_ | | | |
| ICC | 0.68 | | | | 0.44 | | | | 0.63 | | | |
| N | 689 _id_ | | | | 414 _id_ | | | | 383 _id_ | | | |
| Observations | 964 | | | | 476 | | | | 488 | | | |
| Marginal R^2^ / Conditional R^2^ | 0.111 / 0.719 | | | | 0.082 / 0.485 | | | | 0.166 / 0.689 | | | |

**Table S15: Moderate Versus Large Weight Loss analysis: Regression outputs of the association between a change in BMI and a change in HbA1c (mmol/mol) in the non-diabetic normoglycemia population divided by magnitude of weight loss.**

|  | **All Loss** | | | | **BMI loss < 5%** | | | | **BMI loss >= 5%** | | | |
| --- | --- | --- | --- | --- | --- | --- | --- | --- | --- | --- | --- | --- |
| *Coeffcient* | *Estimates* | *std. Error* | *CI (95%)* | *p* | *Estimates* | *std. Error* | *CI (95%)* | *p* | *Estimates* | *std. Error* | *CI (95%)* | *p* |
| Intercept | 8.005 | 2.311 | 3.461 – 12.548 | **0.001** | 0.594 | 3.574 | -6.471 – 7.659 | 0.868 | 12.854 | 2.640 | 7.650 – 18.057 | **<0.001** |
| BMI Change | 0.224 | 0.049 | 0.128 – 0.321 | **<0.001** | 0.782 | 0.379 | 0.032 – 1.532 | **0.041** | 0.142 | 0.074 | -0.003 – 0.287 | 0.055 |
| Baseline Age | 0.033 | 0.014 | 0.006 – 0.060 | **0.016** | 0.005 | 0.020 | -0.034 – 0.045 | 0.785 | 0.048 | 0.016 | 0.017 – 0.079 | **0.003** |
| Gender (Female) | -0.489 | 0.325 | -1.128 – 0.150 | 0.134 | -0.879 | 0.431 | -1.731 – -0.028 | **0.043** | -0.380 | 0.410 | -1.188 – 0.429 | 0.356 |
| Ethnicity (White) | -0.691 | 0.733 | -2.131 – 0.750 | 0.346 | 1.665 | 0.950 | -0.213 – 3.544 | 0.082 | -2.751 | 0.949 | -4.620 – -0.881 | **0.004** |
| Baseline BMI | 0.012 | 0.033 | -0.053 – 0.077 | 0.714 | 0.079 | 0.047 | -0.013 – 0.171 | 0.090 | -0.042 | 0.042 | -0.124 – 0.040 | 0.316 |
| Baseline HbA1c | -0.260 | 0.057 | -0.371 – -0.148 | **<0.001** | -0.117 | 0.087 | -0.289 – 0.054 | 0.177 | -0.326 | 0.065 | -0.454 – -0.197 | **<0.001** |
| Time Point (60 months) | 0.147 | 0.190 | -0.226 – 0.520 | 0.440 | -0.002 | 0.343 | -0.679 – 0.676 | 0.996 | 0.454 | 0.282 | -0.102 – 1.009 | 0.109 |
| **Random Effects** | | | | | | | | | | | | |
| σ^2^ | 2.20 | | | | 1.55 | | | | 2.61 | | | |
| τ_00_ | 4.12 _id_ | | | | 4.37 _id_ | | | | 3.29 _id_ | | | |
| ICC | 0.65 | | | | 0.74 | | | | 0.56 | | | |
| N | 272 _id_ | | | | 141 _id_ | | | | 182 _id_ | | | |
| Observations | 379 | | | | 153 | | | | 226 | | | |
| Marginal R^2^ / Conditional R^2^ | 0.124 / 0.695 | | | | 0.089 / 0.761 | | | | 0.194 / 0.643 | | | |

**Table S16: Moderate Versus Large Weight Loss analysis: Regression outputs of the association between a change in BMI and a change in HbA1c (mmol/mol) in the non-diabetic hyperglycaemia population divided by magnitude of weight loss.**

|  | **All Loss** | | | | **BMI loss < 5%** | | | | **BMI loss >= 5%** | | | |
| --- | --- | --- | --- | --- | --- | --- | --- | --- | --- | --- | --- | --- |
| *Coeffcient* | *Estimates* | *std. Error* | *CI (95%)* | *p* | *Estimates* | *std. Error* | *CI (95%)* | *p* | *Estimates* | *std. Error* | *CI (95%)* | *p* |
| Intercept | 18.591 | 8.464 | 1.788 – 35.394 | **0.030** | 19.441 | 14.410 | -9.601 – 48.483 | 0.184 | 30.141 | 11.041 | 7.843 – 52.440 | **0.009** |
| BMI Change | 0.405 | 0.125 | 0.157 – 0.654 | **0.002** | 0.168 | 0.954 | -1.755 – 2.091 | 0.861 | 0.217 | 0.170 | -0.125 – 0.560 | 0.207 |
| Baseline Age | 0.077 | 0.033 | 0.011 – 0.143 | **0.023** | 0.099 | 0.045 | 0.007 – 0.191 | **0.035** | 0.063 | 0.052 | -0.042 – 0.168 | 0.235 |
| Gender (Female) | -0.818 | 0.628 | -2.065 – 0.429 | 0.196 | -1.508 | 0.927 | -3.376 – 0.361 | 0.111 | -1.291 | 0.951 | -3.212 – 0.630 | 0.182 |
| Ethnicity (White) | -1.350 | 1.035 | -3.404 – 0.704 | 0.195 | -0.635 | 1.429 | -3.515 – 2.244 | 0.659 | -3.078 | 1.648 | -6.407 – 0.250 | 0.069 |
| Baseline BMI | 0.087 | 0.061 | -0.035 – 0.209 | 0.160 | 0.017 | 0.108 | -0.200 – 0.234 | 0.878 | 0.111 | 0.086 | -0.063 – 0.285 | 0.207 |
| Baseline HbA1c | -0.602 | 0.192 | -0.984 – -0.220 | **0.002** | -0.596 | 0.315 | -1.231 – 0.039 | 0.065 | -0.849 | 0.249 | -1.353 – -0.346 | **0.001** |
| Time Point (60 months) | 0.492 | 0.526 | -0.551 – 1.536 | 0.351 | 0.311 | 0.525 | -0.746 – 1.368 | 0.556 | 0.732 | 0.555 | -0.388 – 1.852 | 0.194 |
| **Random Effects** | | | | | | | | | | | | |
| σ^2^ | 5.98 | | | | 0.94 | | | | 1.57 | | | |
| τ_00_ | 1.73 _id_ | | | | 8.34 _id_ | | | | 5.26 _id_ | | | |
| ICC | 0.22 | | | | 0.90 | | | | 0.77 | | | |
| N | 75 _id_ | | | | 49 _id_ | | | | 43 _id_ | | | |
| Observations | 105 | | | | 54 | | | | 51 | | | |
| Marginal R^2^ / Conditional R^2^ | 0.267 / 0.432 | | | | 0.157 / 0.914 | | | | 0.338 / 0.847 | | | |

**Table S17: Moderate Versus Large Weight Loss analysis: Regression outputs of the association between a change in BMI and a change in HbA1c (mmol/mol) in the T2D population divided by magnitude of weight loss.**

|  | **All Loss** | | | | **BMI loss < 5%** | | | | **BMI loss >= 5%** | | | |
| --- | --- | --- | --- | --- | --- | --- | --- | --- | --- | --- | --- | --- |
| *Coeffcient* | *Estimates* | *std. Error* | *CI (95%)* | *p* | *Estimates* | *std. Error* | *CI (95%)* | *p* | *Estimates* | *std. Error* | *CI (95%)* | *p* |
| Intercept | 16.052 | 7.195 | 1.913 – 30.191 | **0.026** | 12.327 | 9.562 | -6.502 – 31.156 | 0.198 | 12.804 | 10.093 | -7.100 – 32.707 | 0.206 |
| BMI Change | 1.487 | 0.264 | 0.968 – 2.006 | **<0.001** | -0.020 | 1.462 | -2.898 – 2.859 | 0.989 | 1.710 | 0.372 | 0.976 – 2.444 | **<0.001** |
| Baseline Age | -0.037 | 0.055 | -0.145 – 0.071 | 0.497 | -0.067 | 0.069 | -0.203 – 0.069 | 0.331 | 0.005 | 0.080 | -0.152 – 0.162 | 0.953 |
| Gender (Female) | 1.279 | 1.316 | -1.306 – 3.864 | 0.331 | 1.284 | 1.649 | -1.963 – 4.531 | 0.437 | 2.694 | 1.908 | -1.069 – 6.456 | 0.160 |
| Ethnicity (White) | 1.493 | 2.081 | -2.597 – 5.582 | 0.474 | 1.742 | 2.415 | -3.014 – 6.498 | 0.471 | 2.684 | 3.306 | -3.835 – 9.203 | 0.418 |
| Baseline BMI | 0.040 | 0.107 | -0.171 – 0.251 | 0.710 | 0.018 | 0.148 | -0.273 – 0.310 | 0.901 | 0.028 | 0.147 | -0.261 – 0.318 | 0.847 |
| Baseline HbA1c | -0.333 | 0.052 | -0.435 – -0.231 | **<0.001** | -0.321 | 0.065 | -0.450 – -0.193 | **<0.001** | -0.338 | 0.078 | -0.492 – -0.184 | **<0.001** |
| Trial | 0.532 | 1.740 | -2.888 – 3.952 | 0.760 | 2.783 | 2.551 | -2.241 – 7.806 | 0.276 | 0.464 | 2.311 | -4.093 – 5.021 | 0.841 |
| Time Point (60 months) | 7.410 | 1.475 | 4.511 – 10.308 | **<0.001** | 8.498 | 2.671 | 3.239 – 13.758 | **0.002** | 10.288 | 2.313 | 5.727 – 14.849 | **<0.001** |
| Time Point (6 months) | -0.825 | 0.846 | -2.488 – 0.837 | 0.330 | -0.852 | 1.333 | -3.476 – 1.772 | 0.523 | -1.659 | 1.339 | -4.300 – 0.981 | 0.217 |
| **Random Effects** | | | | | | | | | | | | |
| σ^2^ | 44.09 | | | | 52.02 | | | | 45.24 | | | |
| τ_00_ | 107.11 _id_ | | | | 99.47 _id_ | | | | 97.86 _id_ | | | |
| ICC | 0.71 | | | | 0.66 | | | | 0.68 | | | |
| N | 342 _id_ | | | | 224 _id_ | | | | 158 _id_ | | | |
| Observations | 480 | | | | 269 | | | | 211 | | | |
| Marginal R^2^ / Conditional R^2^ | 0.166 / 0.757 | | | | 0.125 / 0.700 | | | | 0.219 / 0.753 | | | |

| **Table S18: Controlling for Diabetes, Antihypertensive, and Statin Medication: Regression outputs of the association between a change in BMI and a change in HbA1c (mmol/mol), systolic blood pressure (mmHg) and cholesterol (mmol/L) in a population with type 2 diabetes from the GLoW trial, including medication indicators.** | | | | | | | | | | | | |
| --- | --- | --- | --- | --- | --- | --- | --- | --- | --- | --- | --- | --- |
|  | **HbA1c (mmol/mol)** | | | | **Systolic Blood Pressure (mmHg)** | | | | **Cholesterol (mmol/L)** | | | |
| *Coeffcient* | *Estimates* | *std. Error* | *CI (95%)* | *p* | *Estimates* | *std. Error* | *CI (95%)* | *p* | *Estimates* | *std. Error* | *CI (95%)* | *p* |
| Intercept | 18.864 | 6.440 | 6.206 – 31.523 | **0.004** | 59.810 | 11.064 | 38.024 – 81.596 | **<0.001** | 1.819 | 2.317 | -2.749 – 6.386 | 0.433 |
| BMI Change | 1.508 | 0.239 | 1.037 – 1.979 | **<0.001** | 1.040 | 0.493 | 0.071 – 2.010 | **0.036** | -0.109 | 0.112 | -0.330 – 0.112 | 0.332 |
| Baseline Age | -0.068 | 0.061 | -0.189 – 0.052 | 0.264 | -0.111 | 0.092 | -0.291 – 0.070 | 0.228 | 0.010 | 0.019 | -0.027 – 0.047 | 0.612 |
| Gender (Female) | 2.356 | 1.305 | -0.209 – 4.920 | 0.072 | 0.020 | 1.980 | -3.880 – 3.919 | 0.992 | -0.125 | 0.433 | -0.978 – 0.727 | 0.773 |
| Ethnicity (White) | 3.293 | 2.004 | -0.647 – 7.233 | 0.101 | 7.857 | 3.590 | 0.788 – 14.926 | **0.030** | 0.334 | 0.892 | -1.423 – 2.091 | 0.708 |
| Baseline BMI | 0.036 | 0.100 | -0.161 – 0.233 | 0.721 | 0.267 | 0.173 | -0.073 – 0.608 | 0.124 | 0.012 | 0.039 | -0.064 – 0.089 | 0.749 |
| Baseline HbA1c | -0.339 | 0.053 | -0.442 – -0.235 | **<0.001** |  |  |  |  |  |  |  |  |
| Diabetes Medication | -0.360 | 1.151 | -2.621 – 1.902 | 0.755 | -2.049 | 1.883 | -5.756 – 1.658 | 0.277 | -0.395 | 0.412 | -1.207 – 0.417 | 0.339 |
| Hypertension Medication | -1.331 | 1.269 | -3.825 – 1.164 | 0.295 | 1.950 | 2.083 | -2.152 – 6.051 | 0.350 | -0.768 | 0.460 | -1.675 – 0.139 | 0.097 |
| Hypercholesterolemia Medication | 0.183 | 1.235 | -2.245 – 2.612 | 0.882 | 1.559 | 2.054 | -2.486 – 5.605 | 0.449 | -0.051 | 0.498 | -1.033 – 0.930 | 0.918 |
| Time Point (6 months) | -1.765 | 0.597 | -2.939 – -0.591 | **0.003** | -0.206 | 1.485 | -3.130 – 2.717 | 0.890 | -0.494 | 0.401 | -1.286 – 0.297 | 0.220 |
| Baseline Systolic Blood Pressure |  |  |  |  | -0.520 | 0.052 | -0.623 – -0.417 | **<0.001** |  |  |  |  |
| Baseline Cholesterol |  |  |  |  |  |  |  |  | -0.416 | 0.209 | -0.828 – -0.004 | **0.048** |
| **Random Effects** | | | | | | | | | | | | |
| σ^2^ | 26.10 | | | | 113.03 | | | | 8.46 | | | |
| τ_00_ | 95.27 _id_ | | | | 81.75 _id_ | | | | 0.19 _id_ | | | |
| ICC | 0.78 | | | | 0.42 | | | | 0.02 | | | |
| N | 294 _id_ | | | | 205 _id_ | | | | 177 _id_ | | | |
| Observations | 426 | | | | 275 | | | | 229 | | | |
| Marginal R^2^ / Conditional R^2^ | 0.217 / 0.832 | | | | 0.344 / 0.619 | | | | 0.044 / 0.065 | | | |

| **Table S19: BMI Classification analysis: Regression outputs of the association between BMI change and HbA1c (mmol/mol) in the pooled population divided by BMI classification.** | | | | | | | | | | | | |
| --- | --- | --- | --- | --- | --- | --- | --- | --- | --- | --- | --- | --- |
|  | **Overweight (<30kg/m^2^)** | | | | **Obese (>=30kg/m^2^, <40kg/m^2^)** | | | | **Severely Obese (>=40kg/m^2^)** | | | |
| *Coeffcient* | *Estimates* | *std. Error* | *CI (95%)* | *p* | *Estimates* | *std. Error* | *CI (95%)* | *p* | *Estimates* | *std. Error* | *CI (95%)* | *p* |
| Intercept | -18.658 | 13.715 | -45.652 – 8.336 | 0.175 | 1.884 | 4.656 | -7.253 – 11.022 | 0.686 | 3.331 | 8.950 | -14.342 – 21.004 | 0.710 |
| BMI Change | 0.215 | 0.295 | -0.365 – 0.795 | 0.466 | 0.549 | 0.108 | 0.337 – 0.762 | **<0.001** | 0.980 | 0.162 | 0.661 – 1.300 | **<0.001** |
| Baseline Age | -0.164 | 0.051 | -0.264 – -0.063 | **0.002** | 0.045 | 0.027 | -0.008 – 0.097 | 0.093 | 0.041 | 0.072 | -0.101 – 0.182 | 0.571 |
| Gender (Female) | 1.096 | 1.078 | -1.026 – 3.217 | 0.310 | -0.285 | 0.685 | -1.630 – 1.059 | 0.677 | -0.810 | 1.826 | -4.415 – 2.795 | 0.658 |
| Ethnicity (White) | 4.110 | 1.717 | 0.730 – 7.489 | **0.017** | -2.382 | 1.328 | -4.988 – 0.224 | 0.073 | 3.864 | 3.956 | -3.948 – 11.676 | 0.330 |
| Baseline HbA1c | -0.056 | 0.063 | -0.181 – 0.068 | 0.372 | -0.294 | 0.032 | -0.357 – -0.232 | **<0.001** | -0.414 | 0.064 | -0.540 – -0.288 | **<0.001** |
| Baseline BMI | 0.692 | 0.437 | -0.168 – 1.552 | 0.114 | 0.181 | 0.126 | -0.067 – 0.429 | 0.152 | 0.084 | 0.132 | -0.178 – 0.346 | 0.527 |
| Trial | 5.582 | 1.747 | 2.143 – 9.021 | **0.002** | 3.706 | 0.898 | 1.943 – 5.469 | **<0.001** | 5.936 | 2.026 | 1.935 – 9.936 | **0.004** |
| Time Point (60 months) | 1.688 | 1.105 | -0.486 – 3.862 | 0.128 | 1.039 | 0.554 | -0.049 – 2.127 | 0.061 | 0.379 | 1.301 | -2.190 – 2.947 | 0.771 |
| Time Point (6 months) | -1.872 | 1.105 | -4.047 – 0.304 | 0.091 | -2.372 | 0.744 | -3.831 – -0.913 | **0.001** | 0.239 | 1.312 | -2.352 – 2.830 | 0.856 |
| **Random Effects** | | | | | | | | | | | | |
| σ^2^ | 37.04 | | | | 36.43 | | | | 28.95 | | | |
| τ_00_ | 27.62 _id_ | | | | 35.55 _id_ | | | | 49.31 _id_ | | | |
| ICC | 0.43 | | | | 0.49 | | | | 0.63 | | | |
| N | 181 _id_ | | | | 583 _id_ | | | | 114 _id_ | | | |
| Observations | 298 | | | | 921 | | | | 174 | | | |
| Marginal R^2^ / Conditional R^2^ | 0.096 / 0.482 | | | | 0.147 / 0.568 | | | | 0.393 / 0.775 | | | |

**Table S20: Sensitivity Analysis: Regression Outputs associating a change in BMI change to a change in HbA1c (mmol/mol) between trial populations with T2D**

|  | **T2D - WRAP** | | | | **T2D - GLOW** | | | |
| --- | --- | --- | --- | --- | --- | --- | --- | --- |
| *Coeffcient* | *Estimates* | *std. Error* | *CI (95%)* | *p* | *Estimates* | *std. Error* | *CI (95%)* | *p* |
| Intercept | 15.887 | 13.487 | -10.782 – 42.557 | 0.241 | 21.641 | 5.027 | 11.766 – 31.515 | **<0.001** |
| BMI Change | 1.294 | 0.469 | 0.366 – 2.223 | **0.007** | 1.636 | 0.207 | 1.229 – 2.044 | **<0.001** |
| Baseline Age | 0.051 | 0.112 | -0.171 – 0.272 | 0.651 | -0.104 | 0.046 | -0.194 – -0.014 | **0.024** |
| Gender (Female) | -2.252 | 2.725 | -7.641 – 3.138 | 0.410 | 1.558 | 1.063 | -0.530 – 3.647 | 0.143 |
| Ethnicity (White) | 0.499 | 4.197 | -7.800 – 8.797 | 0.906 | 2.050 | 1.577 | -1.049 – 5.148 | 0.194 |
| Baseline BMI | -0.052 | 0.279 | -0.605 – 0.500 | 0.852 | 0.121 | 0.085 | -0.045 – 0.287 | 0.154 |
| Baseline HbA1c | -0.327 | 0.100 | -0.525 – -0.130 | **0.001** | -0.389 | 0.044 | -0.474 – -0.303 | **<0.001** |
| Time Point (60 months) | 6.576 | 2.132 | 2.360 – 10.792 | **0.002** |  |  |  |  |
| Time Point (6 months) |  |  |  |  | -1.686 | 0.536 | -2.739 – -0.633 | **0.002** |
| **Random Effects** | | | | | | | | |
| σ^2^ | 153.70 | | | | 31.32 | | | |
| τ_00_ | 57.41 _id_ | | | | 76.13 _id_ | | | |
| ICC | 0.27 | | | | 0.71 | | | |
| N | 90 _id_ | | | | 366 _id_ | | | |
| Observations | 147 | | | | 562 | | | |
| Marginal R^2^ / Conditional R^2^ | 0.207 / 0.422 | | | | 0.253 / 0.782 | | | |

**Table S21: Sensitivity Analysis: Regression outputs associating a change in BMI change to a change in metabolic risk factor in the whole population with diabetes indicator and interaction term**

|  | **HbA1c (mmol/mol)** | | | | **Cholesterol (mmol/L)** | | | | **Systolic Blood Pressure (mmHg)** | | | |
| --- | --- | --- | --- | --- | --- | --- | --- | --- | --- | --- | --- | --- |
| *Coeffcient* | *Estimates* | *std. Error* | *CI (95%)* | *p* | *Estimates* | *std. Error* | *CI (95%)* | *p* | *Estimates* | *std. Error* | *CI (95%)* | *p* |
| Intercept | 8.934 | 2.596 | 3.841 – 14.027 | **0.001** | 1.160 | 0.264 | 0.643 – 1.678 | **<0.001** | 52.133 | 4.442 | 43.419 – 60.847 | **<0.001** |
| BMI Change | 0.216 | 0.112 | -0.004 – 0.435 | 0.055 | 0.036 | 0.010 | 0.017 – 0.055 | **<0.001** | 0.831 | 0.172 | 0.494 – 1.168 | **<0.001** |
| Baseline Age | -0.019 | 0.021 | -0.061 – 0.022 | 0.363 | -0.001 | 0.002 | -0.005 – 0.003 | 0.561 | 0.065 | 0.035 | -0.004 – 0.134 | 0.063 |
| Gender (Female) | 0.030 | 0.528 | -1.007 – 1.067 | 0.955 | 0.229 | 0.051 | 0.130 – 0.329 | **<0.001** | -2.129 | 0.832 | -3.761 – -0.496 | **0.011** |
| Ethnicity (White) | 1.029 | 0.899 | -0.733 – 2.792 | 0.252 | 0.124 | 0.101 | -0.075 – 0.322 | 0.222 | 1.958 | 1.592 | -1.166 – 5.082 | 0.219 |
| Baseline BMI | 0.098 | 0.047 | 0.006 – 0.190 | **0.037** | 0.000 | 0.005 | -0.009 – 0.010 | 0.955 | 0.145 | 0.078 | -0.008 – 0.298 | 0.063 |
| Baseline HbA1c | -0.343 | 0.028 | -0.398 – -0.288 | **<0.001** |  |  |  |  |  |  |  |  |
| NDH | 1.583 | 0.977 | -0.334 – 3.500 | 0.105 | -0.054 | 0.081 | -0.212 – 0.105 | 0.505 | 0.521 | 1.405 | -2.236 – 3.278 | 0.711 |
| T2D | 8.235 | 1.067 | 6.142 – 10.327 | **<0.001** | -0.116 | 0.083 | -0.278 – 0.046 | 0.159 | 2.133 | 1.240 | -0.300 – 4.565 | 0.086 |
| Trial | -0.349 | 0.960 | -2.232 – 1.533 | 0.716 | 0.102 | 0.097 | -0.089 – 0.293 | 0.295 | 1.218 | 1.581 | -1.883 – 4.319 | 0.441 |
| Time Point (60 months) | 1.525 | 0.465 | 0.613 – 2.437 | **0.001** | -0.046 | 0.042 | -0.128 – 0.036 | 0.267 | 1.481 | 0.769 | -0.027 – 2.989 | 0.054 |
| Time Point (6 months) | -1.746 | 0.549 | -2.823 – -0.668 | **0.002** | 0.037 | 0.077 | -0.115 – 0.189 | 0.631 | -0.795 | 1.399 | -3.539 – 1.949 | 0.570 |
| NDH * BMI Change | 0.245 | 0.245 | -0.235 – 0.725 | 0.317 | -0.019 | 0.021 | -0.060 – 0.022 | 0.370 | 0.188 | 0.382 | -0.563 – 0.938 | 0.624 |
| T2D * BMI Change | 1.325 | 0.180 | 0.971 – 1.678 | **<0.001** | -0.014 | 0.019 | -0.050 – 0.022 | 0.450 | 0.478 | 0.311 | -0.132 – 1.088 | 0.124 |
| Baseline Cholesterol |  |  |  |  | -0.317 | 0.023 | -0.361 – -0.272 | **<0.001** |  |  |  |  |
| Baseline SBP |  |  |  |  |  |  |  |  | -0.485 | 0.023 | -0.531 – -0.440 | **<0.001** |
| **Random Effects** | | | | | | | | | | | | |
| σ^2^ | 35.98 | | | | 0.29 | | | | 128.93 | | | |
| τ_00_ | 31.54 _id_ | | | | 0.19 _id_ | | | | 40.97 _id_ | | | |
| ICC | 0.47 | | | | 0.39 | | | | 0.24 | | | |
| N | 894 _id_ | | | | 724 _id_ | | | | 849 _id_ | | | |
| Observations | 1416 | | | | 1100 | | | | 1337 | | | |
| Marginal R^2^ / Conditional R^2^ | 0.210 / 0.579 | | | | 0.206 / 0.515 | | | | 0.335 / 0.495 | | | |

**Table S22: Sensitivity Analysis: Base Analysis with Multiple Imputation pooled regression outputs associating a change in BMI change to a change in HbA1c (mmol/mol).**

| **Characteristic** | **All** | | | | **NDN** | | | | **NDH** | | | | **T2D** | | | |  |
| --- | --- | --- | --- | --- | --- | --- | --- | --- | --- | --- | --- | --- | --- | --- | --- | --- | --- |
|  | **Beta** | **SE***^1^* | **95% CI***^1^* | **p-value** | **Beta** | **SE***^1^* | **95% CI***^1^* | **p-value** | **Beta** | **SE***^1^* | **95% CI***^1^* | **p-value** | **Beta** | **SE***^1^* | **95% CI***^1^* | **p-value** | |
| BMI Change | 0.44 | 0.055 | 0.33, 0.55 | <0.001 | 0.30 | 0.026 | 0.24, 0.35 | <0.001 | 0.57 | 0.090 | 0.39, 0.75 | <0.001 | 0.98 | 0.139 | 0.70, 1.3 | <0.001 | |
| Baseline Age | 0.01 | 0.012 | -0.01, 0.03 | 0.4 | 0.02 | 0.008 | 0.00, 0.04 | 0.019 | 0.04 | 0.026 | -0.01, 0.09 | 0.10 | -0.05 | 0.029 | -0.11, 0.01 | 0.093 | |
| Gender (Female) | -0.23 | 0.328 | -0.88, 0.42 | 0.5 | -0.57 | 0.207 | -0.98, -0.17 | 0.006 | -1.3 | 0.605 | -2.5, -0.10 | 0.034 | 0.16 | 0.642 | -1.1, 1.4 | 0.8 | |
| Ethnicity (white) | 0.28 | 0.612 | -0.95, 1.5 | 0.7 | -0.51 | 0.548 | -1.6, 0.59 | 0.4 | 0.05 | 0.936 | -1.8, 1.9 | >0.9 | 1.2 | 0.915 | -0.60, 3.0 | 0.2 | |
| Baseline BMI | 0.08 | 0.030 | 0.03, 0.14 | 0.005 | 0.05 | 0.021 | 0.01, 0.09 | 0.024 | 0.18 | 0.057 | 0.07, 0.29 | 0.002 | 0.09 | 0.053 | -0.01, 0.20 | 0.084 | |
| Baseline HbA1c | -0.22 | 0.024 | -0.27, -0.17 | <0.001 | -0.19 | 0.036 | -0.26, -0.11 | <0.001 | -0.29 | 0.212 | -0.71, 0.13 | 0.2 | -0.28 | 0.031 | -0.34, -0.21 | <0.001 | |
| Trial | 2.4 | 0.466 | 1.4, 3.3 | <0.001 |  |  |  |  |  |  |  |  | -0.73 | 0.819 | -2.4, 0.90 | 0.4 | |
| Time Point (24 months) | -0.06 | 0.253 | -0.56, 0.44 | 0.8 | 0.02 | 0.144 | -0.26, 0.31 | 0.9 | 0.13 | 0.441 | -0.75, 1.0 | 0.8 | -0.19 | 0.440 | -1.1, 0.68 | 0.7 | |
| Time Point (60 months) | -0.03 | 0.228 | -0.48, 0.42 | >0.9 | 0.14 | 0.171 | -0.20, 0.48 | 0.4 | 0.30 | 0.421 | -0.54, 1.1 | 0.5 | 0.01 | 0.482 | -0.94, 0.97 | >0.9 | |
| Time Point (6 months) | -0.26 | 0.241 | -0.74, 0.22 | 0.3 | 0.08 | 0.157 | -0.24, 0.39 | 0.6 | 0.01 | 0.475 | -0.93, 0.96 | >0.9 | -0.63 | 0.481 | -1.6, 0.32 | 0.2 | |
| *^1^* SE = Standard Error, CI = Confidence Interval | | | | | | | | | | | | | | | | |  |

**Table S23: Sensitivity Analysis: Base Analysis with Multiple Imputation pooled regression outputs associating a change in BMI change to a change in Cholesterol (mmol/L).**

| **Characteristic** | **All** | | | | **NDN** | | | | | **NDH** | | | | | **T2D** | | | |  |
| --- | --- | --- | --- | --- | --- | --- | --- | --- | --- | --- | --- | --- | --- | --- | --- | --- | --- | --- | --- |
|  | **Beta** | **SE***^1^* | **95% CI***^1^* | **p-value** | | **Beta** | **SE***^1^* | **95% CI***^1^* | **p-value** | | **Beta** | **SE***^1^* | **95% CI***^1^* | **p-value** | **Beta** | **SE***^1^* | **95% CI***^1^* | **p-value** | |
| BMI Change | 0.01 | 0.019 | -0.03, 0.05 | 0.5 | | 0.03 | 0.010 | 0.01, 0.05 | 0.001 | | 0.01 | 0.019 | -0.02, 0.05 | 0.5 | 0.00 | 0.041 | -0.09, 0.08 | >0.9 | |
| Baseline Age | 0.00 | 0.004 | -0.01, 0.01 | 0.7 | | 0.00 | 0.003 | 0.00, 0.01 | 0.6 | | 0.00 | 0.007 | -0.01, 0.02 | 0.9 | 0.00 | 0.008 | -0.02, 0.01 | 0.6 | |
| Gender (Female) | 0.06 | 0.085 | -0.11, 0.23 | 0.5 | | 0.26 | 0.058 | 0.15, 0.38 | <0.001 | | 0.16 | 0.158 | -0.16, 0.48 | 0.3 | -0.11 | 0.190 | -0.49, 0.27 | 0.6 | |
| Ethnicity (white) | 0.21 | 0.138 | -0.06, 0.49 | 0.13 | | 0.20 | 0.133 | -0.07, 0.46 | 0.15 | | 0.06 | 0.241 | -0.43, 0.55 | 0.8 | 0.03 | 0.265 | -0.50, 0.56 | >0.9 | |
| Baseline BMI | -0.01 | 0.008 | -0.02, 0.01 | 0.5 | | 0.00 | 0.006 | -0.01, 0.01 | 0.7 | | -0.01 | 0.013 | -0.04, 0.01 | 0.3 | 0.00 | 0.019 | -0.04, 0.04 | 0.9 | |
| Baseline HbA1c | -0.34 | 0.064 | -0.47, -0.20 | <0.001 | | -0.32 | 0.026 | -0.38, -0.27 | <0.001 | | -0.29 | 0.053 | -0.40, -0.19 | <0.001 | -0.23 | 0.116 | -0.47, 0.01 | 0.061 | |
| Trial | 0.16 | 0.097 | -0.04, 0.35 | 0.11 | |  |  |  |  | |  |  |  |  | 0.23 | 0.217 | -0.20, 0.67 | 0.3 | |
| Time Point (24 months) | -0.04 | 0.066 | -0.17, 0.09 | 0.5 | | 0.00 | 0.048 | -0.10, 0.10 | >0.9 | | -0.06 | 0.100 | -0.26, 0.14 | 0.6 | -0.04 | 0.190 | -0.42, 0.34 | 0.8 | |
| Time Point (60 months) | -0.04 | 0.070 | -0.18, 0.10 | 0.5 | | -0.01 | 0.046 | -0.10, 0.09 | 0.9 | | -0.10 | 0.087 | -0.27, 0.07 | 0.3 | -0.02 | 0.167 | -0.35, 0.32 | >0.9 | |
| Time Point (6 months) | -0.03 | 0.068 | -0.16, 0.11 | 0.7 | | 0.00 | 0.057 | -0.12, 0.11 | >0.9 | | -0.05 | 0.106 | -0.26, 0.16 | 0.6 | -0.06 | 0.144 | -0.35, 0.22 | 0.7 | |
| *^1^* SE = Standard Error, CI = Confidence Interval | | | | | | | | | | | | | | | | | | |  |

**Table S24: Sensitivity Analysis: Base Analysis with Multiple Imputation pooled regression outputs associating a change in BMI change to a change in Systolic Blood Pressure (mmHg).**

| **Characteristic** | **All** | | | | **NDN** | | | | | **NDH** | | | | **T2D** | | |  |
| --- | --- | --- | --- | --- | --- | --- | --- | --- | --- | --- | --- | --- | --- | --- | --- | --- | --- |
|  | **Beta** | **SE***^1^* | **95% CI***^1^* | **p-value** | | **Beta** | **SE***^1^* | **95% CI***^1^* | **p-value** | **Beta** | **SE***^1^* | **95% CI***^1^* | **p-value** | **Beta** | **SE***^1^* | **95% CI***^1^* | **p-value** |
| BMI Change | 0.92 | 0.089 | 0.74, 1.1 | <0.001 | | 0.97 | 0.157 | 0.65, 1.3 | <0.001 | 1.1 | 0.377 | 0.38, 1.9 | 0.004 | 1.1 | 0.240 | 0.63, 1.6 | <0.001 |
| Baseline Age | 0.11 | 0.024 | 0.06, 0.16 | <0.001 | | 0.09 | 0.038 | 0.01, 0.16 | 0.022 | 0.04 | 0.131 | -0.22, 0.30 | 0.8 | 0.04 | 0.045 | -0.05, 0.13 | 0.4 |
| Gender (Female) | -2.0 | 0.626 | -3.2, -0.71 | 0.002 | | -1.1 | 1.08 | -3.3, 1.0 | 0.3 | -3.9 | 2.75 | -9.4, 1.6 | 0.2 | -2.4 | 0.930 | -4.2, -0.53 | 0.012 |
| Ethnicity (white) | 0.23 | 1.05 | -1.9, 2.3 | 0.8 | | -2.0 | 2.21 | -6.4, 2.3 | 0.4 | 1.6 | 3.56 | -5.5, 8.7 | 0.7 | 2.3 | 1.79 | -1.4, 5.9 | 0.2 |
| Baseline BMI | 0.20 | 0.063 | 0.07, 0.32 | 0.003 | | 0.04 | 0.097 | -0.15, 0.23 | 0.7 | 0.47 | 0.220 | 0.03, 0.91 | 0.035 | 0.18 | 0.101 | -0.02, 0.39 | 0.077 |
| Baseline HbA1c | -0.51 | 0.019 | -0.55, -0.47 | <0.001 | | -0.39 | 0.032 | -0.45, -0.33 | <0.001 | -0.55 | 0.067 | -0.68, -0.42 | <0.001 | -0.59 | 0.028 | -0.64, -0.53 | <0.001 |
| Trial | 1.4 | 0.666 | 0.11, 2.8 | 0.034 | |  |  |  |  |  |  |  |  | 0.00 | 1.11 | -2.2, 2.2 | >0.9 |
| Time Point (24 months) | 0.13 | 0.503 | -0.87, 1.1 | 0.8 | | 0.74 | 0.725 | -0.70, 2.2 | 0.3 | -1.4 | 1.75 | -4.8, 2.1 | 0.4 | 0.09 | 0.816 | -1.5, 1.7 | >0.9 |
| Time Point (60 months) | 0.28 | 0.423 | -0.56, 1.1 | 0.5 | | 1.6 | 0.728 | 0.15, 3.0 | 0.031 | -1.8 | 1.64 | -5.0, 1.5 | 0.3 | -0.06 | 0.788 | -1.6, 1.5 | >0.9 |
| Time Point (6 months) | 0.05 | 0.488 | -0.92, 1.0 | >0.9 | | 0.86 | 0.783 | -0.70, 2.4 | 0.3 | -0.87 | 2.16 | -5.2, 3.5 | 0.7 | -0.36 | 0.735 | -1.8, 1.1 | 0.6 |
| *^1^* SE = Standard Error, CI = Confidence Interval | | | | | | | | | | | | | | | | |  |


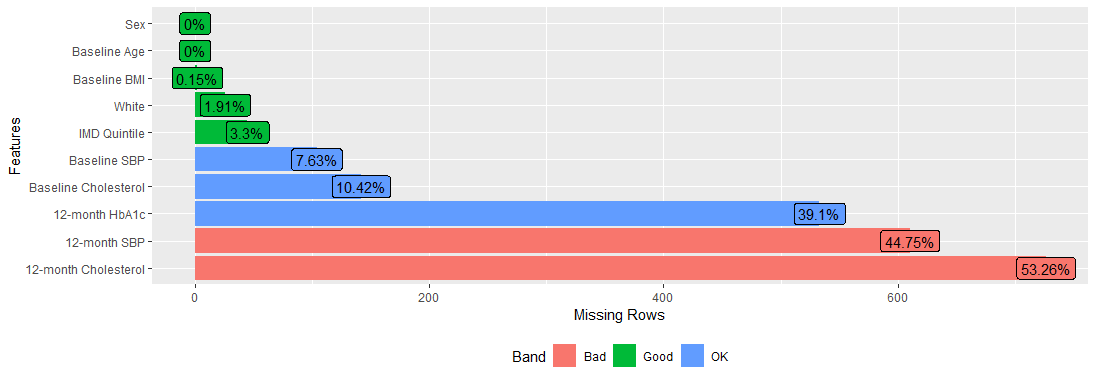


**Figure S1: Percentage of rows with missing data in pooled data with available baseline HbA1c.** Multiple Imputation with chained equations was completed using the mice package in R, implementing the predictive mean matching method. All variables in the regression analyses were included, in addition to IMD quintile. The subgroups: individuals with normoglycemia, individuals with non-diabetic hyperglycaemia, and individuals with type 2 diabetes were imputed separately. Twenty imputations, with 10 iterations were deemed appropriate after a visual check of convergence of measures was performed. Regression estimates were pooled assuming Rubin’s Rules.
