# Supplementary material for: Effects of weight loss and weight gain on HbA1c, systolic blood pressure and total cholesterol in three subgroups defined by blood glucose: a pooled analysis of two behavioural weight management trials in England
Source: BMJ Open. 2025 Apr 15;15(4):e095046. doi: 10.1136/bmjopen-2024-095046 (PMC12004498; doi:10.1136/bmjopen-2024-095046)
Supplement: Supplementary file 2 [file bmjopen-2024-095046.reviewer_comments.pdf]

PEER REVIEW HISTORY

BMJ Open publishes all reviews undertaken for accepted manuscripts. Reviewers are asked to complete a checklist review form and are provided with free text boxes to elaborate on their assessment. These free text comments are reproduced below.

This paper was submitted to another journal from BMJ but declined for publication following peer review. The authors addressed the reviewers' comments and submitted the revised paper to BMJ Open. The paper was subsequently accepted for publication at BMJ Open.

ARTICLE DETAILS

Title (Provisional)

The effects of weight loss and weight gain on HbA1c, systolic blood pressure and total cholesterol in three subgroups defined by blood glucose: a pooled analysis of two behavioural weight management trials in England.

Authors

Pidd, Katharine; Breeze, Penny; Ahern, Amy; Griffin, Simon J; Brennan, Alan

---

VERSION 1 - REVIEW

**Reviewer**	**1**
**Name**	**Reviewer 1**
**Affiliation**	
**Date**	**26-Aug-2024**
**COI**	

Comments not published pending reviewer's consent

VERSION 2 - REVIEW

**Reviewer**	**2**
**Name**	**Fiorina, Paolo**
**Affiliation**	**University of Milan**
**Date**	**04-Dec-2024**
**COI**	**None**

This study is a well conducted pooled analysis of patients data from two behavioural weight management trials (WRAP and GLoW) whose main goal is to search for an association

between weight changes and HbA1c level, Total Cholesterol and Systolic Blood Pressure (SBP). Patients were divided into three groups:

a. HbA1c < 42 mmol/mol -> Normoglycaemic patients

b. HbA1c =42-48 -> Non Diabetic Hyperglycaemic patients

c. HbA1c > 48 or a known diagnosis of diabetes -> Diabetic patients

Using linear mixed-effects regressions and while controlling for confounders like age at baseline, sex, ethnicity, BMI at baseline, baseline independent variable reading and time point or measurement within the trial, authors demonstrate that

- Weight change has an impact on HbA1c level, particularly within the subgroup of patients with baseline diabetes

- Weight gain has a larger impact on HbA1c than weight loss, particularly in noroglycaemic patients

- Weight loss associates with total cholesterol and SBP improvements

The main novelty element of this study is related to the apparently stronger impact of weight gain on HbA1c of normoglycaemic patients as the effects of weight loss and weight gain in diabetes has already been extensively addressed in the past (PMID: 28417575, PMID: 23796131). Moreover the beneficial effects of weight loss on the lipid profile have already been investigated in previous studies (PMID: 23796131, PMID: 33686615) although in a somewhat different population. In particular in a study from Montefusco L et al. (PMID: 33686615) weight loss is demonstrated to improve the inflammatory cytokine profile that in turn may play a major role in improving CVD risk. Nevertheless, the evidence provided strengthens previous knowledge and add new insights on weight management benefits paticularly in the non-diabetic population.

There are some issues that might be worthwhile to address on the manuscript:

MAIN COMMENTS

#1- In the “statistical approach” paragraph, Authors report that “Control variables included within the regression as fixed effects were age at baseline, sex, ethnicity, BMI at baseline, baseline observation for the independent variable, time-point and trial sample for all regressions”. As patients included are mostly women aged around 50 yo and having the menopause state an impact both on weight and on cardiometabolic risk, it would be of use, if available, to include data, at least in the supplementary material, about the proportion of patients entering menopause before or during the study and investigating whether this variable acts as a confounder on HbA1c, SPB and cholesterol

#2- Smoke status or smoke cessation during the study might have an influence on body weight changes. For instance, quitting smoking during the study follow up might limit or impair weight loss however the benefits of quitting smoke clearly overweight the effects of

weight on global cardiovascular risk. Are there any data available on patients smoking status and particularly on the smoke quitting rate throughout the study?

#3- Table 2 and 3 is formally perfectly correct, however it might result hard to read for clinicians. Could it be possible to make it more reader friendly? For instance a possible approach could be to change “Coef.” with a title referring to the variable measured (i.e. HbA1c or SBP or Cholesterol) and “Obs.” with “n”

#4- Are there any data on LDL cholesterol in addition to total cholesterol? As LDL would be a better predictor of CVD.

MINOR COMMENTS:

#5- In the abstract’s paragraph “What this study adds” it is reported that “HbA1c changes more with weight in those with non-diabetic normoglycemia than in those with normoglycaemia”. I suppose the first “normoglycaemia” should instead be “hyperglycaemia”. Could you please verify and possibly correct?

#6- Consider adding in the discussion a mention on the potential role of weight loss in improving the inflammatory status as previously shown in the aforementioned study by Montefusco L. ed al. (PMID: 33686615)

#7- The text reported in the last part of the “Outcome measures” paragraph within the METHODS section, from page 6 line 53 of the revised manuscript (i.e. “Baseline characteristics for the trials….”) up to the end of the “Outcome measures” paragraph including table 1 report more results than actual methods. Therefore I would consider moving this part to the RESULTS section.

#8- In the paragraph “Strengths and limitations”, page 18 line 49 of the R1 manuscript it is reported that TSD is “associated with reduce control of blood pressure” it should probably be “reduced” instead

#9- Throughout the paper and in figure S1 patients are defined as “white individuals”. I suggest to consider changing “white individuals” to something like “Caucasian Ethnicity” or similar.

**Reviewer**	**3**
**Name**	**Kelson, Zoe**
**Affiliation**	**University of Exeter, Mathematics**
**Date**	**31-Dec-2024**
**COI**	**None**

This study estimates the association between weight and cardiometabolic risk factors across subgroups of individuals with normoglycaemia, nondiabetic hyperglycaemia, and type 2

diabetes (T2D), and explores whether the association differs between weight loss and weight gain.

Reviewer comments:

"Individual participant data from the Weight loss Referral for Adults in Primary care (WRAP, n=1267) and Glucose Lowering through Weight management (GLoW, n=577) trials were pooled"

and

"The sample population was divided into three subgroups: those with normoglycaemia, those with non-diabetic hyperglycaemia and those with T2D"

and

"In both trials, over 80% of participants were white, and majority were women"

The authors satisfactorily comment on the representativeness of the trials in the discussion of the study limitations.

"Data from two behavioural weight management randomised control trials were pooled to form the sample."

and

"The proportion of individuals by Index of Multiple Deprivation was unbalanced between the trials, however it was balanced in the combined sample. The average baseline BMI and SBP did not differ between the trials, however, average HbA1c was higher in the GLoW trial, due to difference in inclusion criteria, and average cholesterol was higher in the WRAP trial"

The authors suitably adjust for trial as a control variable in the analyses.

"Linear mixed-effects regressions estimated the relationships between a change in body mass index (BMI; kg/m 2) and a change in glycated haemoglobin (HbA1c; mmol/mol), total cholesterol (mmol/L) or systolic blood pressure (SBP; mmHg)" [Abstract]

and

"The difference in the mean changes between subpopulations was evaluated using a Welch t-test. The primary analysis investigated how a change in BMI related to a change in HbA1c (mmol/mol), total cholesterol (mmol/L) or SBP (mmHg) using a linear mixed-effects regression approach for each subgroup. The dependent variable described change in BMI from baseline to each follow-up point at 6, 12, and 60 months and the independent variables described change in the cardiometabolic risk over the corresponding time point, from baseline to 6, 12 and 60 months. Control variables included within the regression as fixed effects were age at baseline, sex, ethnicity, BMI at baseline, baseline observation for the independent variable, time-point and trial sample for all regressions. "

Appropriate modelling methods have been applied.

"The regressions allow variation in the intercept by participant to account for multiple measures generated across each time point."

To clarify, does the model account for repeated measures by participant at each time point, as well as changes across different time points by participant?

"A complete case analysis was used, assuming that data were missing completely at random"

and

"To assess the impact of missing data on regression outputs, incomplete variables were imputed for individuals with recorded baseline HbA1c using multiple imputation with chained equations (MICE) for the primary analysis. "

The authors do well to conduct a sensitivity analysis on the approach for handling missing data, which helps to demonstrate the robustness of the study findings.

"Additional analyses considered the difference between the relationship between BMI and HBA1c, cholesterol and SBP depending on the direction or magnitude of weight change"

and

"To evaluate the impact of medication use, additional medication indicators were added to the model"

and

"Finally, an exploratory analysis considered the impact of baseline BMI on the relationship between weight change and HbA1c by dividing the pooled population by BMI classifications overweight"

and

"An additional regression with a diabetes status indicator and interaction term with BMI change was performed on the pooled population to see how the relationship changes between subgroups.

To investigate trial differences in the relationship across the T2D population, the base regression analysis between BMI and HbA1c was performed on the WRAP and GLoW populations separately and the regression outputs compared. "

These insightful analyses help to explore patterns and associations in the data, as well as demonstrate the robustness of the study findings.

"Figure 1: Estimated 12-month HbA1c changes in relation to BMI changes."

Can uncertainty (confidence intervals) please be added to this plot?

"generalisability is limited by the demographic composition of the trials, both containing a high proportion of women and white individuals. Although, trial population differences were accommodated for through control variables within the regression analysis. The trials

evaluated group based behavioural weight loss interventions. This limits applicability of the relationship in the context of alternative interventions such as diet restrictions, physical activity, medication, or surgery, which can influence metabolic risk factors differently (6, 20, 24)... Certain pharmacological treatments for hypertension and hypercholesterolemia have been associated with reduced glycaemic control and new onset of T2D (17, 18, 25). T2D is associated with reduce control of blood pressure and cholesterol levels (15, 16). Developments in pharmacological treatments for diabetes and excess weight, such as Semaglutide, (26, 27) may also affect how BMI and HBA1c interact. Further research as to whether these medications change the relationship between BMI and cardiometabolic risk factors may provide important insight."

The authors have suitably discussed the study limitations.

The supplementary material is detailed and informative.

Were any reporting guidelines adhered to that the authors can provide the associated checklist for?

VERSION 2 - AUTHOR RESPONSE

Reviewer 2 Comments:

Comments to the Author:
This study is a well conducted pooled analysis of patients data from two behavioural weight management trials (WRAP and GLoW) whose main goal is to search for an association between weight changes and HbA1c level, Total Cholesterol and Systolic Blood Pressure (SBP). Patients were divided into three groups:
a. HbA1c < 42 mmol/mol -> Normoglycaemic patients
b. HbA1c =42-48 -> Non Diabetic Hyperglycaemic patients
c. HbA1c > 48 or a known diagnosis of diabetes -> Diabetic patients
Using linear mixed-effects regressions and while controlling for confounders like age at baseline, sex, ethnicity, BMI at baseline, baseline independent variable reading and time point or measurement within the trial, authors demonstrate that
- Weight change has an impact on HbA1c level, particularly within the subgroup of patients with baseline diabetes
- Weight gain has a larger impact on HbA1c than weight loss, particularly in noroglycaemic patients
- Weight loss associates with total cholesterol and SBP improvements
The main novelty element of this study is related to the apparently stronger impact of weight gain on HbA1c of normoglycaemic patients as the effects of weight loss and weight gain in diabetes has already been extensively addressed in the past (PMID: 28417575, PMID: 23796131). Moreover the beneficial effects of weight loss on the lipid profile have already been investigated in previous studies (PMID: 23796131, PMID: 33686615) although in a somewhat different population. In particular in a study from Montefusco L et al. (PMID: 33686615) weight loss is demonstrated to improve the inflammatory cytokine profile that in turn may play a major

role in improving CVD risk. Nevertheless, the evidence provided strengthens previous knowledge and add new insights on weight management benefits particularly in the non-diabetic population.

We thank the reviewer for their supportive assessment of the manuscript and appreciate their constructive and thoughtful feedback.

There are some issues that might be worthwhile to address on the manuscript:

MAIN COMMENTS

#1- In the "statistical approach" paragraph, Authors report that "Control variables included within the regression as fixed effects were age at baseline, sex, ethnicity, BMI at baseline, baseline observation for the independent variable, time-point and trial sample for all regressions". As patients included are mostly women aged around 50 yo and having the menopause state an impact both on weight and on cardiometabolic risk, it would be of use, if available, to include data, at least in the supplementary material, about the proportion of patients entering menopause before or during the study and investigating whether this variable acts as a confounder on HbA1c, SPB and cholesterol
#2- Smoke status or smoke cessation during the study might have an influence on body weight changes. For instance, quitting smoking during the study follow up might limit or impair weight loss however the benefits of quitting smoke clearly overweight the effects of weight on global cardiovascular risk. Are there any data available on patients smoking status and particularly on the smoke quitting rate throughout the study?

Unfortunately, due to data availability we are not able to explore how these may impact the way weight change is related to HbA1c, blood pressure and cholesterol levels within this analysis. We agree with the reviewer that both menopause and smoking cessation may have an impact on the relationships investigated within this analysis and recognise that they are an important consideration, given the evidence of their observed impact with weight and metabolic risk factors previously found. A discussion point has been added recognising this as a limitation of our analysis (page17. Discussion: Strengths and limitations).

"Additional population characteristics may impact the relationships estimated. Smoking cessation and menopause have both previously been observed to relate to changes in weight and cardiometabolic risk, however, data availability meant exploration of this was not possible within this analysis."

#3- Table 2 and 3 is formally perfectly correct, however it might result hard to read for clinicians. Could it be possible to make it more reader friendly? For instance a possible approach could be to change "Coef." with a title referring to the variable measured (i.e. HbA1c or SBP or Cholesterol) and "Obs." with "n"

We appreciate this suggestion and recognise the importance of ensuring research is easily accessible. We have updated tables 2 and 3 in line with this recommended.

#4- Are there any data on LDL cholesterol in addition to total cholesterol? As LDL would be a better predictor of CVD.

Unfortunately, data for LDL over time was not available to us for this analysis for WRAP trial population. While this data is available for the GLoW trial population, we felt an additional analysis with only a population with type 2 diabetes (as in the GLoW trial) would add little to this paper given its emphasis on comparing the relationships by diabetes status. A discussion point has been added in the Discussion section of the potential for future analyses relating BMI change to other cardiometabolic risk factors, such as LDL (page17. Discussion: Strengths and limitations).

"While HbA1c, blood pressure and cholesterol levels are important predictors of cardiovascular disease, weight change has been observed to relate to improvements in other metabolic risk factors such as low-density lipoproteins, triglycerides or inflammatory markers (9, 24). Future research could aim to estimate the relationship between BMI change and these other factors across diabetes status subgroups."

MINOR COMMENTS:
#5- In the abstract's paragraph "What this study adds" it is reported that "HbA1c changes more with weight in those with non-diabetic normoglycemia than in those with normoglycaemia". I suppose the first "normoglycaemia" should instead be "hyperglycaemia". Could you please verify and possibly correct?

This section has been removed in response to a request from the editor. Therefore, typo has been removed in the process.

#6- Consider adding in the discussion a mention on the potential role of weight loss in improving the inflammatory status as previously shown in the aforementioned study by Montefusco L. ed al. (PMID: 33686615)

The study mentioned investigates the role of weight loss on improvements in risk factors including inflammatory indicators in the context of caloric restriction. A discussion point highlighting that future research could assess the relationship between weight change and other health indicators, such as inflammatory markers, has been added with this paper referenced as evidence of observed changes in this context (page17. Discussion: Strengths and limitations).

"While HbA1c, blood pressure and cholesterol levels are important predictors of cardiovascular disease, weight change has been observed to relate to improvements in other metabolic risk factors such as low-density lipoproteins, triglycerides or inflammatory markers (9, 24). Future research could aim to estimate the relationship between BMI change and these other factors across diabetes status subgroups."

#7- The text reported in the last part of the "Outcome measures" paragraph within the METHODS section, from page 6 line 53 of the revised manuscript (i.e. "Baseline characteristics

for the trials....”) up to the end of the “Outcome measures” paragraph including table 1 report more results than actual methods. Therefore I would consider moving this part to the RESULTS section.

The description of the baseline population and the summary statistics provided have been moved into the results section.

#8- In the paragraph “Strengths and limitations”, page 18 line 49 of the R1 manuscript it is reported that TSD is “associated with reduce control of blood pressure” it should probably be “reduced” instead

This incorrect spelling has been corrected accordingly.

#9- Throughout the paper and in figure S1 patients are defined as “white individuals”. I suggest to consider changing “white individuals” to something like “Caucasian Ethnicity” or similar.

We appreciate the need to use the correct terminology to describe ethnicity and thank the reviewer for their suggestion here. In this manuscript we use descriptors that are consistent with the UK Government guidelines, which suggest “white people” is an acceptable descriptor. As a result, we have decided to maintain the terminology used.

Reviewer 3 Comments:

Comments to the Author:
This study estimates the association between weight and cardiometabolic risk factors across subgroups of individuals with normoglycaemia, nondiabetic hyperglycaemia, and type 2 diabetes (T2D), and explores whether the association differs between weight loss and weight gain.

Reviewer comments:
"Individual participant data from the Weight loss Referral for Adults in Primary care (WRAP, n=1267) and Glucose Lowering through Weight management (GLoW, n=577) trials were pooled"
and
"The sample population was divided into three subgroups: those with normoglycaemia, those with non-diabetic hyperglycaemia and those with T2D"
and
"In both trials, over 80% of participants were white, and majority were women"

The authors satisfactorily comment on the representativeness of the trials in the discussion of the study limitations.

"Data from two behavioural weight management randomised control trials were pooled to form the sample."
and
"The proportion of individuals by Index of Multiple Deprivation was unbalanced between the trials, however it was balanced in the combined sample. The average baseline BMI and SBP did

not differ between the trials, however, average HbA1c was higher in the GLoW trial, due to difference in inclusion criteria, and average cholesterol was higher in the WRAP trial"
The authors suitably adjust for trial as a control variable in the analyses.

"Linear mixed-effects regressions estimated the relationships between a change in body mass index (BMI; kg/m 2) and a change in glycated haemoglobin (HbA1c; mmol/mol), total cholesterol (mmol/L) or systolic blood pressure (SBP; mmHg)" [Abstract]
and
"The difference in the mean changes between subpopulations was evaluated using a Welch t-test. The primary analysis investigated how a change in BMI related to a change in HbA1c (mmol/mol), total cholesterol (mmol/L) or SBP (mmHg) using a linear mixed-effects regression approach for each subgroup. The dependent variable described change in BMI from baseline to each follow-up point at 6, 12, and 60 months and the independent variables described change in the cardiometabolic risk over the corresponding time point, from baseline to 6, 12 and 60 months. Control variables included within the regression as fixed effects were age at baseline, sex, ethnicity, BMI at baseline, baseline observation for the independent variable, time-point and trial sample for all regressions. "

Appropriate modelling methods have been applied.

We thank the reviewer for their support of our chosen methodology.

"The regressions allow variation in the intercept by participant to account for multiple measures generated across each time point."

To clarify, does the model account for repeated measures by participant at each time point, as well as changes across different time points by participant?

The mixed-effects regression model accounts for individual variability. The text has been updated to clarify this point.

“The regressions allow variation in the intercept by participant.”

"A complete case analysis was used, assuming that data were missing completely at random"
and
"To assess the impact of missing data on regression outputs, incomplete variables were imputed for individuals with recorded baseline HbA1c using multiple imputation with chained equations (MICE) for the primary analysis. "
The authors do well to conduct a sensitivity analysis on the approach for handling missing data, which helps to demonstrate the robustness of the study findings.

"Additional analyses considered the difference between the relationship between BMI and HBA1c, cholesterol and SBP depending on the direction or magnitude of weight change"
and

"To evaluate the impact of medication use, additional medication indicators were added to the model"
and
"Finally, an exploratory analysis considered the impact of baseline BMI on the relationship between weight change and HbA1c by dividing the pooled population by BMI classifications overweight"
and
"An additional regression with a diabetes status indicator and interaction term with BMI change was performed on the pooled population to see how the relationship changes between subgroups.
To investigate trial differences in the relationship across the T2D population, the base regression analysis between BMI and HbA1c was performed on the WRAP and GLoW populations separately and the regression outputs compared. "
These insightful analyses help to explore patterns and associations in the data, as well as demonstrate the robustness of the study findings.

We appreciate the reviewer's recognition of robustness of the results presented.

"Figure 1: Estimated 12-month HbA1c changes in relation to BMI changes."
Can uncertainty (confidence intervals) please be added to this plot?

We have adapted this graph to include uncertainty in the coefficients relating to BMI change, as represented by the example individual. The updated graph is on page 23.

"generalisability is limited by the demographic composition of the trials, both containing a high proportion of women and white individuals. Although, trial population differences were accommodated for through control variables within the regression analysis. The trials evaluated group based behavioural weight loss interventions. This limits applicability of the relationship in the context of alternative interventions such as diet restrictions, physical activity, medication, or surgery, which can influence metabolic risk factors differently (6, 20, 24)... Certain pharmacological treatments for hypertension and hypercholesterolemia have been associated with reduced glycaemic control and new onset of T2D (17, 18, 25). T2D is associated with reduce control of blood pressure and cholesterol levels (15, 16). Developments in pharmacological treatments for diabetes and excess weight, such as Semaglutide, (26, 27) may also affect how BMI and HBA1c interact. Further research as to whether these medications change the relationship between BMI and cardiometabolic risk factors may provide important insight."
The authors have suitably discussed the study limitations.

The supplementary material is detailed and informative.

We thank the reviewer for their positive comments on the manuscript.

Were any reporting guidelines adhered to that the authors can provide the associated checklist for?

To ensure we are following good reporting practices, we have provided a completed STROBE checklist in the resubmission. The manuscript and supplementary materials have been updated to ensure each criterion is met.

VERSION 3 - REVIEW

**Reviewer**	**2**
**Name**	**Fiorina, Paolo**
**Affiliation**	**University of Milan**
**Date**	**27-Feb-2025**
**COI**	

Authors have successfully replied to reviewer's comments and amended accordingly

VERSION 3 - AUTHOR RESPONSE

Reviewer 2 Comments:

Comments to the Author:
Authors have successfully replied to reviewer's comments and amended accordingly.

We thank the reviewer for their valuable comments on this manuscript and we are pleased that previous revisions have met the reviewers' expectations.
